# Supplementary material for: Economic evaluation of the Target-D platform to match depression management to severity prognosis in primary care: A within-trial cost-utility analysis
Source: PLoS One. 2022 May 25;17(5):e0268948. doi: 10.1371/journal.pone.0268948 (PMC9132336; doi:10.1371/journal.pone.0268948)
Supplement: S1 Appendix — (PDF) [file pone.0268948.s001.pdf]

Supplementary Materials

Supplementary Text S1. Detailed description of Target-D interventions

All participants completed a brief eligibility screening survey on an iPad in their GP waiting room and were not required to disclose any information to the research assistant other than their willingness to complete this survey. The eligibility survey was integrated with the Target-D platform, consent form, randomisation schedule and baseline and follow-up measures in a purpose-built website, accessible on any internet-enabled device. As part of the consent process, eligible patients were asked to enter an email address; if they were unable or unwilling to complete the baseline assessment and CPT using the iPad provided in the waiting room, they were emailed a link to do so on their own device at a time that was convenient to them. Research assistants followed up with non-responders via phone, text and/or email.

Intervention arm

After completing the clinical prediction tool, participants randomly allocated to the intervention arm received:

- feedback on their responses;
- an opportunity to set mental health priorities and reflect on the importance of addressing these priorities and their confidence in doing so; and
- a management option matched to their predicted depressive symptom severity.

Together, these elements comprise the Target-D platform. The presentation of the platform was informed by the principles of motivational interviewing,[1] a psychologically-driven goal modelling approach,[2] and developed with input from end-users.[3] The CPT comprises the PHQ-9 plus eight additional items assessing sex, anxiety, general health, living situation and financial security. These additional items, as well as providing some predictive power over and above that provided by the PHQ-9, are included in recognition of the broader determinants of poor mental health. By taking a holistic approach to mental health rather than considering depressive symptoms alone, the intervention aims allow people to set priorities and engage with care options that are relevant to their needs. Recommended management options were displayed on screen immediately after completing the CPT and re-iterated in follow-up contact from the Target-D team (as described below). All participants also received an automated email encouraging them to speak with their GP regarding any concerns they may have about their mental health and providing contact details for community-based services (e.g., crisis support lines). Selected management options had RCT evidence of effectiveness for the appropriate level of depressive symptom severity, as described

below; management and planned follow-up procedures for each prognostic group are described below.

Minimal/mild prognostic group

Participants in this group were recommended to use the myCompass program, an online, CBT-based, self-help resource comprising information, treatment modules, homework activities and mood tracking functions.[4] At the time of this study, information and mood tracking functions could be accessed on any internet-enabled device, although the treatment modules were computer-based. Target-D participants were free to use myCompass as much or as little as they liked. They received an initial welcome email from the Target-D team providing the link to myCompass with a brief outline of what to expect on first log in and a follow-up telephone call from a research assistant to discuss their treatment recommendation and troubleshoot if needed. Up to four attempts at this call were made. Finally, participants in this group were sent an email one week after completing the CPT (or after all call attempts were exhausted) reminding them of the benefits of myCompass and encouraging them to register for the program if they hadn't already. Adherence was defined as completion of at least one module, as indicated by website analytics provided by the Black Dog Institute (who manage the myCompass program).

Moderate prognostic group

Participants in this group received a recommendation to use the This Way Up iCBT program (specifically, the Worry and Sadness course); a guided, linear program comprising six online lessons, homework activities and symptom monitoring.[5] Participants were free to complete as many or as few lessons as they wished and to access the course when, where and using the device that was convenient to them. They received an initial email from the study team with information about the program and advising that they would receive a separate email from This Way Up with a unique link providing them with free access to the course for 90 days.

Research assistants then contacted participants weekly via phone or email either until they completed Lesson Two or until four weeks after they were emailed their unique link, whichever came first. One phone call attempt was made at each scheduled contact, with a personalised email sent to non-responders (tailored to their progress through the program). When participants reported a worsening of depressive symptoms within This Way Up (≥ 5 points on the PHQ-9 from their previous assessment), an automated email was generated to both the Target-D team and the participant encouraging the participant to access further support. Adherence was defined as

completion of all 6 lessons in the Worry and Sadness course, as indicated by website analytics provided by the This Way Up team at the University of New South Wales.

Severe prognostic group

Participants in this group were offered collaborative care,[6-9] described on the Target-D platform as an opportunity to work together with a specially trained nurse and their GP to identify options to improve their emotional health and wellbeing. Participants were offered up to eight structured appointments with the nurse over 12 weeks. The intervention aimed to improve outcomes by supporting participants' engagement in and ownership over their own health care by applying the principles of motivational interviewing.[1]

A research assistant contacted participants allocated to this group via phone to discuss their treatment recommendation and schedule their first appointment with a Target-D nurse. Four call attempts were made, after which the participant was emailed a brief introduction to the collaborative care intervention and invited to get in touch with the study team to schedule an appointment. Participants were reminded of subsequent appointments via SMS from their Target-D nurse and could contact their nurse directly via SMS or phone to reschedule as required.

The collaborative care intervention was delivered by five female registered nurses with between 13 and 21 years of experience in a range of fields including primary, emergency and intensive care nursing. All nurses completed a 2-day training course on the background to Target-D and trial protocol (day 1; delivered by project manager) and an introduction to motivational interviewing techniques (day 2; delivered by registered psychologist). Nurses were assisted to put these techniques into practice through detailed procedure manuals and structured appointment templates which stepped through the process of developing a plan to improve participants' mental health. The template for appointment 1 was pre-populated with the priority areas the participant identified in the Target-D platform. This provided structure to their first interaction with the Target-D nurse and established a focus for the collaborative care intervention across the eight appointments, as follows:

- Appointment 1: reflect on current situation, set goals relevant to each priority area and identify actions they could take to meet those goals.
- Appointments 2 – 7: review progress, identify barriers to taking action and how these may be overcome
- Appointment 8: review progress and identify additional supports required or actions to take after Target-D.

In order to facilitate rapport building, participants were encouraged to attend appointments in person at their general practice (particularly the first appointment) but this was not a requirement and they were free to meet with their nurse either over the phone or in person, according to individual preference. After each appointment, the Target-D nurse provided the participant with a copy of their plan (via email or in hard copy) to remind and support them with taking their intended actions that week. The nurse also provided a copy of the plan to the participant’s GP and other professionals involved in the participant’s mental health care. In supporting participants to develop their plan, Target-D nurses spent time outside the eight structured appointments to research appropriate services both within and external to the health system, discuss management options with GPs and other professionals and draft referrals for GPs. Target-D nurses were also able to contact both the project manager and registered psychologist for support and guidance as required; no additional strategies were employed to encourage fidelity to intervention delivery.

Adherence was defined as completion of eight appointments, as indicated by appointment logs completed by the nurses delivering the intervention. Nurse fidelity to the collaborative care model was assessed through review of written plans and appointment logs and of audio recordings conducted for a subset of appointments. This data are currently being analysed and will be reported separately.

Control arm

After completing the CPT, participants randomly allocated to the control arm did not receive symptom feedback, priority setting, or prognosis-matched treatment recommendations. Instead, they received usual care plus Target-D attention control (UC+) in the form of a telephone call from a trained research assistant to reiterate the importance of involvement in the trial, address questions and concerns as required and administer a brief structured interview about research participation. Up to four attempts at contacting participants via phone were made, after which an email was sent encouraging the participant to contact the study team. All participants in this arm also received the automated email sent to the intervention arm providing information about community-based services and encouragement to speak to their GP about mental health concerns. They were free to continue accessing health services as usual throughout the duration of the trial.

Supplementary Table S2. Impact inventory as recommended by the Second Panel on Cost-Effectiveness in Health and Medicine

Sector	Type of impact	Included in this reference case analysis from … perspective?		Notes on sources of evidence
		Health sector	Societal	
Formal Health Care Sector				
Health	*Health outcomes (effects)*			
	Longevity effects			
	Health-related quality-of-life effects	✓	✓	AQoL-8D
	Other health effects (eg, adverse events and secondary transmissions of infections)			
	Medical costs			
	Paid for by third-party payers	✓	✓	Medications, consultations, hospital care reimbursed by government
	Paid for by patients out-of-pocket	✓	✓	Gap fees for medications, consultations, hospital care
	Future related medical costs (payers and patients)			
	Future unrelated medical costs (payers and patients)			
Informal Health Care Sector				
Health	Patient-time costs	NA		
	Unpaid caregiver-time costs	NA		
	Transportation costs	NA		
Non-Health Care Sectors (with examples of possible items)				
Productivity	Labour market earnings lost	NA	✓	Self-reported paid work loss
	Cost of unpaid lost productivity due to illness	NA	✓	Self-reported unpaid work loss
	Cost of uncompensated household production	NA		
Consumption	Future consumption unrelated to health	NA		
Social Services	Cost of social services as part of intervention	NA		

Sector	Type of impact	Included in this reference case analysis from … perspective?		Notes on sources of evidence
		Health sector	Societal	
Non-Health Care Sectors (with examples of possible items) (*continued)*				
Legal or criminal justice	Number of crimes related to intervention	NA		
	Cost of crimes related to intervention	NA		
Education	Impact of intervention on educational achievement of population	NA		
Housing	Cost of intervention on home improvements (eg, removing lead paint)	NA		
Environment	Production of toxic waste or pollution by intervention	NA		
Other (specify)		NA		

Table adapted from: Sanders et al. [10]

Supplementary Table S3. Description of intervention costing

Item	Unit cost	Unit	Quantity	Total Cost	Data sources and assumptions
**Screening phase (n = 45,615)**					
Implementation and maintenance of the clinical prediction tool (CPT)				$10,000	Routine data collected for the Target-D RCT.
iPads to undertake CPT	$533.76	Per iPad	6	$3,203	Assume outright purchase, no maintenance and in new condition at the end of 1 year with a 5% discount rate on resale price.
Wi-Fi dongles for screening	$51.82	Per dongle	3	$155	Weighted average cost of the 3 dongles attributable to the screening phase.
Time spent by research assistants (RAs) to approach patients in GP waiting room	$39.81	Per hour	760	$30,266	Assumes each encounter takes 1 minute of RA time. Assume casual RA wage rate incorporates on-costs.
Total cost of screening (excluding sunk costs)				*$43,624*	Base case excludes research and development costs.
Average health sector cost per person for those invited to complete screening questionnaire (base case - excluding sunk costs)			45,615	**$0.96**	**Base case.** Applied to all participants in the intervention arm (but not the control arm).
Research and development of the CPT				$61,423	Routine data collected for the Target-D RCT.
Total cost of screening (including sunk costs)				*$105,046*	Sensitivity analysis includes research and development costs.
Average health sector cost per person for those invited to complete screening questionnaire (sensitivity analysis - including sunk costs)			45,615	**$2.30**	**Sensitivity analysis.** Applied to all participants in the intervention arm (but not the control arm).

Item	Unit cost	Unit	Quantity	Total Cost	Data sources and assumptions
**Mild prognostic group (n = 679)**					
Check-in phone call	$39.81	Per hour	43	$1,718	Assume each call takes 5 minutes of RA time. Assume casual RA wage rate incorporates on-costs.
Registration to the myCompass online program	$60.74	Per person	123	$7,471	Annual cost of myCompass online program derived from budgeted delivery costs per user.[11]
Total cost among mild prognostic group				*$9,189*	
Average health sector cost per person among the mild prognostic group (base case)			679	**$14**	**Base case.** Applied to participants in the mild prognostic group of the intervention arm.
**Moderate prognostic group (n = 143)**					
Check-in phone call	$39.81	Per hour	12	$464	Assume each call takes 5 minutes of RA time. Assume casual RA wage rate incorporates on-costs.
Registration to the ThisWayUp 'Worry and Sadness' iCBT course †	$238.89	Per person	70	$16,722	Average between ThisWayUp user fee (A$56.00) and MindSpot Wellbeing Course annual cost (A$421.77).[12,13]
Progress reviews - Weeks 1 to 4	$39.81	Per hour	42	$1,689	Assume each call takes 5 minutes of RA time. Assume casual RA wage rate incorporates on-costs.
Total cost among moderate prognostic group in base case analysis				$18,875	Base case uses average unit cost between ThisWayUp and MindSpot Wellbeing Course ($239.89).
Average health sector cost per person among the moderate prognostic group (base case)			143	$132	**Base case.** Applied to participants in the moderate prognostic group of the intervention arm.
Total cost among moderate prognostic group in sensitivity analysis				$31,677	Sensitivity analysis uses highest unit cost involving MindSpot Wellbeing Course (A$421.77).
Average health sector cost per person among the moderate prognostic group (sensitivity analysis)			143	$222	**Sensitivity analysis.** Applied to participants in the moderate prognostic group of the intervention arm.

Item	Unit cost	Unit	Quantity	Total Cost	Data sources and assumptions
**Severe prognostic group (n = 111)**					
Check-in phone call	$39.81	Per hour	6	$249	Assume each call takes 5 minutes of RA time. Assume casual RA wage rate incorporates on-costs.
Laptops for research nurses	$1,348	Per laptop	4	$5,394	Assumes outright purchase, no maintenance and in new condition at the end of 1 year with a 5% discount rate on resale price.
Mobile phones for research nurses	$60.38	Per mobile	4	$242	Assumes outright purchase, no maintenance and no resale value.
Mobile phone credit for research nurses	$292.00	Per person	5	$1,460	
Wi-Fi dongles for research nurses	$40.51	Per dongle	3	$122	Weighted average cost of the three dongles attributable to the intervention phase.
Research nurse training - catering (one-day session)	$15.00	Per person	6	$90	Five care navigators underwent training, plus the trainer.
Research nurse training - cost of the trainer (clinical psychologist)	$1,278	Per day	3	$3,833	Clinical psychologist trainer paid A$1278 per day. They ran training session twice due to staff turnover.
Research nurse time	$50.88	Per hour	1,250	$63,606	100% of time recorded by research nurses counted as Target-D care navigation time. They were employed casually and only worked hours delivering care.
Total cost among severe prognostic group				$74,995	
Average health sector cost per person among the severe prognostic group (base case)			111	$676	**Base case.** Applied to participants in the severe prognostic group of the intervention arm.

† The true cost of the ThisWayUp 'Worry and Sadness' iCBT course is unknown. Patients are charged a nominal fee of A$56 which does not reflect the opportunity cost of the intervention. This fee is charged to incentivise program completion rather than to cover program costs. True cost will be higher.

Supplementary Text S4. Methods to cost health service use

Diagnostic tests and the majority of health professional visits were costed using a weighted average cost paid by the government for the specific service as derived from the Medicare Benefits Schedule (MBS) item reports.[14] In cases where health professionals do not receive reimbursement through an MBS item (e.g., counsellors, alcohol and drug workers), costs were estimated using an hourly wage rate (plus 30% on-costs).[15] Community services were costed using the national average hourly wage rate (plus 30% on-costs).[15] Pharmaceutical Benefits Scheme (PBS) item prices were used to calculate the government and patient out-of-pocket costs for government-subsidised medications reported in the resource use questionnaire.[16] Online Australian retail pharmacy sites were used to cost medications and supplements that are not covered by the PBS. Hospitalisations were costed using the national average cost of an acute admission to a public hospital from the 2016/2017 National Hospital Cost Data Collection,[17] while emergency department visits used a national average cost derived from the same report.[17] All unit costs were adjusted for inflation and converted to 2018/2019 values using the total health price index.[18] The national average hourly wage rate (plus 30% on-costs) was used to value lost work time.[15] Time taken off from unpaid work was valued at 25% of the value of lost work time to reflect the value of lost leisure time.[19]

Supplementary Text S5. Analysis of missing data mechanisms

The mechanisms underlying missing responses for utility scores (i.e., health outcomes) and cost variables were analysed by: (1) investigating overall patterns of missing data; and (2) performing multivariate logistic regression analyses to determine if data missingness was related to other observed variables collected at baseline.

A summary of the top ten missing data patterns for utility and cost variables, at 3-month and 12-month follow-up, is presented in *Table S5.1*. Complete data were observed across 33% of study participants. A monotone missing pattern, which typically involves missing data caused by loss to follow up, was observed across 55% of participants. Of these, 37% had missing data across both follow-up periods and 18% had missing data at 12-month follow-up only. A non-monotone missing pattern, where missing cases at 3-month follow-up provided subsequent data at 12-month follow-up, was observed across 6% of participants. Miscellaneous missing data patterns were observed across the remaining 6% of participants.

Table S5.1 Summary of missing data patterns at 3-month and 12-month follow-up

	Missing-value patterns (1 means complete)													
	3-month variables							12-month variables						
Percent	Utility - AQoL-8D score	Cost - Health professional visits	Cost - Emergency department visits	Cost - Overnight hospitalisations	Cost - Diagnostic tests	Cost - Medications	Cost - Productivity losses	Utility - AQoL-8D score	Cost - Health professional visits	Cost - Emergency department visits	Cost - Overnight hospitalisations	Cost - Diagnostic tests	Cost - Medications	Cost - Productivity losses
33%	1	1	1	1	1	1	1	1	1	1	1	1	1	1
37%	0	0	0	0	0	0	0	0	0	0	0	0	0	0
18%	1	1	1	1	1	1	1	0	0	0	0	0	0	0
6%	0	0	0	0	0	0	0	1	1	1	1	1	1	1
1%	1	1	1	1	1	1	1	0	1	0	0	0	0	0
1%	0	1	0	0	0	0	0	0	0	0	0	0	0	0
<1%	0	1	1	1	1	1	1	0	0	0	0	0	0	0
<1%	1	1	1	1	1	1	1	0	1	1	1	1	1	1
<1%	0	0	0	0	0	0	0	0	1	0	0	0	0	0
<1%	0	0	0	0	0	0	0	0	1	1	1	1	1	1

Based on the missing data patterns outlined above, there is evidence to suggest that the underlying missing data mechanism for utility and cost variables is, at a minimum, due to data being missing at random. Data would be considered missing not at random if there was a systematic relationship between the value of the missing variable and the likelihood of a missing response (e.g., participants with a high income level are less likely to provide responses to variables asking about an individual's income level). Among 94% of participants, missing responses were equally likely across utility and cost variables, regardless of the follow-up period. If data were missing not at random then it is expected that there would be an unequal probability of missing responses between the utility and cost variables, as responses to questions about utility (i.e., health-related quality of life) will be qualitatively different to questions involving health care expenditure and/or productivity losses. Moreover, there is no *a priori* reason to believe that there is a systematic relationship underlying participant non-response, such that utility and cost data were missing not at random.

A variable is considered to have values that are missing at random when missing responses are systematically related to other observed variables. Multivariate logistic regressions were performed to analyse whether a missing response to a utility/cost variable was associated with any of the sociodemographic variables collected at baseline. These baseline sociodemographic variables included: the trial arm, gender, clinic, prognostic group, age, PHQ-9 score, highest level of education, current employment status, health care card status, long term illness, self-rated health, living alone, ability to manage on available income, having visited a psychiatrist/counsellor in the past 12 months and current use of antidepressants.

The results of the logistic regression analysing the relationship between missing utility responses and baseline variables are presented in *Table S5.2*. Trial arm, gender, clinic, age, highest level of education and having visited a psychiatrist/counsellor in the past 12 months were all significantly associated with missing utility responses. The results of the logistic regression analysing the relationship between missing cost responses and baseline variables are presented in *Table S5.3*. Trial arm, clinic, age and having visited a psychiatrist/counsellor in the past 12 months were all significantly associated with missing cost responses. The results of the multivariate logistic regressions described above provide further evidence that missing utility/cost data are missing at random, given that missing responses were found to be associated with other sociodemographic variables collected at baseline.

Table S5.2 Results of the multivariate logistic regression model analysing the relationship between missing utility values and baseline sociodemographic variables

Covariate	Odds ratio	Std. error	95% CI	p-value
Trial arm=Control (base level)	1.00	0.00		
Trial arm=Intervention	1.68	0.18	[1.36, 2.07]	<0.01 *
Gender=Male (base level)	1.00	0.00		
Gender=Female	0.77	0.09	[0.61, 0.97]	0.026 *
Gender=Other	0.21	0.16	[0.05, 0.97]	0.046 *
Clinic=1 (base level)	1.00	0.00		
Clinic=2	2.18	1.12	[0.80, 5.96]	0.130
Clinic=3	1.45	0.89	[0.44, 4.81]	0.542
Clinic=4	1.66	0.88	[0.59, 4.70]	0.338
Clinic=5	3.22	1.59	[1.22, 8.49]	0.018 *
Clinic=6	3.48	2.04	[1.10,11.00]	0.034 *
Clinic=7	3.41	1.71	[1.27, 9.13]	0.015 *
Clinic=8	3.92	2.33	[1.23,12.55]	0.021 *
Clinic=9	11.26	5.89	[4.04,31.40]	<0.01 *
Clinic=10	1.88	0.96	[0.70, 5.09]	0.212
Clinic=11	7.35	3.79	[2.67,20.21]	<0.01 *
Clinic=12	5.27	2.61	[1.99,13.91]	<0.01 *
Clinic=13	5.35	2.70	[1.99,14.40]	<0.01 *
Clinic=14	5.48	3.07	[1.83,16.41]	<0.01 *
Prognostic group=Mild (base level)	1.00	0.00		
Prognostic group=Moderate	1.30	0.26	[0.88, 1.93]	0.188
Prognostic group=Severe	1.67	0.50	[0.93, 3.00]	0.086
Age (range: 18-65 years)	0.98	0.01	[0.97, 0.99]	<0.01 *
Depression – PHQ-9 score (range: 0-27)	0.98	0.02	[0.95, 1.02]	0.344
Education=Year 11 or lower (base level)	1.00	0.00		
Education=Year 12	0.91	0.20	[0.59, 1.39]	0.652
Education=Certificate/diploma	0.68	0.13	[0.46, 0.99]	0.045 *
Education=Bachelor degree or higher	0.67	0.13	[0.46, 0.97]	0.036 *
Employment=Employed/working (base level)	1.00	0.00		
Employment=Unemployed and looking for work	1.34	0.25	[0.93, 1.94]	0.116
Employment=Neither working nor looking for work	1.17	0.19	[0.85, 1.59]	0.336
No Health Care Card=No (base level)	1.00	0.00		
No Health Care Card=Yes	1.18	0.16	[0.90, 1.54]	0.238
Long term Illness=No (base level)	1.00	0.00		
Long term Illness=Yes	1.05	0.15	[0.80, 1.38]	0.728
Self-rated health=Excellent (base level)	1.00	0.00		
Self-rated health=Very good	1.20	0.29	[0.75, 1.94]	0.442
Self-rated health=Good	1.18	0.28	[0.73, 1.88]	0.498
Self-rated health=Fair	1.17	0.32	[0.69, 2.00]	0.552
Self-rated health=Poor	0.77	0.27	[0.39, 1.53]	0.454
Live alone=No (base level)	1.00	0.00		
Live alone=Yes	0.92	0.15	[0.67, 1.26]	0.597
Manage on available income=Easily/Not too bad (base level)	1.00	0.00		
Manage on available income=Difficult some of the time	0.99	0.18	[0.70, 1.42]	0.974
Visited psychologist/counsellor in 12 months=No (base level)	1.00	0.00		
Visited psychologist/counsellor in 12 months=Yes	0.71	0.08	[0.57, 0.89]	<0.01 *
Antidepressant=No (base level)	1.00	0.00		
Antidepressant=Yes	0.96	0.13	[0.73, 1.25]	0.737
Intercept	1.34	0.85	[0.39, 4.61]	0.638

** p-value less than 0.05*

Table S5.3 Results of the multivariate logistic regression model analysing the relationship between missing cost values and baseline sociodemographic variables

Covariate	Odds ratio	Std. error	95% CI	p-value
Trial arm=Control (base level)	1.00	0.00		
Trial arm=Intervention	1.70	0.18	[1.39, 2.09]	<0.01 *
Gender=Male (base level)	1.00	0.00		
Gender=Female	0.84	0.10	[0.67, 1.05]	0.120
Gender=Other	0.26	0.20	[0.06, 1.19]	0.083
Clinic=1 (base level)	1.00	0.00		
Clinic=2	2.11	1.08	[0.77, 5.77]	0.146
Clinic=3	1.40	0.85	[0.42, 4.63]	0.581
Clinic=4	1.79	0.95	[0.64, 5.06]	0.269
Clinic=5	2.99	1.48	[1.13, 7.88]	0.027 *
Clinic=6	3.26	1.91	[1.03,10.29]	0.044 *
Clinic=7	3.37	1.69	[1.26, 9.02]	0.016 *
Clinic=8	3.81	2.26	[1.19,12.16]	0.024 *
Clinic=9	10.71	5.58	[3.85,29.75]	<0.01 *
Clinic=10	1.78	0.90	[0.66, 4.82]	0.255
Clinic=11	5.52	2.82	[2.03,15.02]	<0.01 *
Clinic=12	4.33	2.14	[1.64,11.39]	<0.01 *
Clinic=13	3.92	1.97	[1.47,10.50]	<0.01 *
Clinic=14	4.91	2.73	[1.65,14.60]	<0.01 *
Prognostic group=Mild (base level)	1.00	0.00		
Prognostic group=Moderate	1.24	0.24	[0.85, 1.83]	0.269
Prognostic group=Severe	1.40	0.41	[0.79, 2.48]	0.244
Age (range: 18-65 years)	0.98	0.01	[0.97, 0.99]	<0.01 *
Depression – PHQ-9 score (0-27)	0.99	0.02	[0.96, 1.02]	0.514
Education=Year 11 or lower (base level)	1.00	0.00		
Education=Year 12	0.99	0.21	[0.65, 1.51]	0.974
Education=Certificate/diploma	0.80	0.15	[0.55, 1.16]	0.241
Education=Bachelor degree or higher	0.72	0.14	[0.50, 1.04]	0.079
Employment=Employed/working (base level)	1.00	0.00		
Employment=Unemployed and looking for work	1.28	0.23	[0.90, 1.82]	0.177
Employment=Neither working nor looking for work	1.17	0.18	[0.86, 1.58]	0.316
No Health Care Card=No (base level)	1.00	0.00		
No Health Care Card=Yes	1.01	0.13	[0.77, 1.31]	0.966
Long term Illness=No (base level)	1.00	0.00		
Long term Illness=Yes	1.00	0.13	[0.77, 1.31]	0.979
Self-rated health=Excellent (base level)	1.00	0.00		
Self-rated health=Very good	1.19	0.28	[0.75, 1.90]	0.452
Self-rated health=Good	1.10	0.26	[0.70, 1.75]	0.672
Self-rated health=Fair	1.15	0.31	[0.69, 1.94]	0.592
Self-rated health=Poor	0.80	0.28	[0.41, 1.57]	0.524
Live alone=No (base level)	1.00	0.00		
Live alone=Yes	1.10	0.18	[0.81, 1.52]	0.537
Manage on available income=Easily/Not too bad (base level)	1.00	0.00		
Manage on available income=Difficult some of the time	0.98	0.17	[0.69, 1.38]	0.910
Visited psychologist/counsellor in 12 months=No (base level)	1.00	0.00		
Visited psychologist/counsellor in 12 months=Yes	0.70	0.08	[0.57, 0.87]	<0.01 *
Antidepressant=No (base level)	1.00	0.00		
Antidepressant=Yes	0.99	0.13	[0.76, 1.28]	0.915
Intercept	1.28	0.79	[0.38, 4.32]	0.696

** p-value less than 0.05*

In summary, there is reasonable evidence to suggest that missing utility/cost data can be considered missing at random. The use of multiple imputation as a means of addressing missing data is valid when the underlying missing data mechanism is, at a minimum, deemed missing at random. If data were missing not at random, then this would lead to biased statistical inferences. The current study has incorporated the multiple imputation method to deal with missing utility/cost data as part of the statistical analysis. Based on the logistic regression analyses presented above, multiple imputation methods were used to impute missing utility/cost data, with adjustment for the following baseline covariates that were associated with missing utility/cost values: trial arm, gender, clinic, age, highest level of education and having visited a psychiatrist/counsellor in the past 12 months.

Supplementary Figure S6. Cost-effectiveness results for the health sector perspective across all participants at 3 months

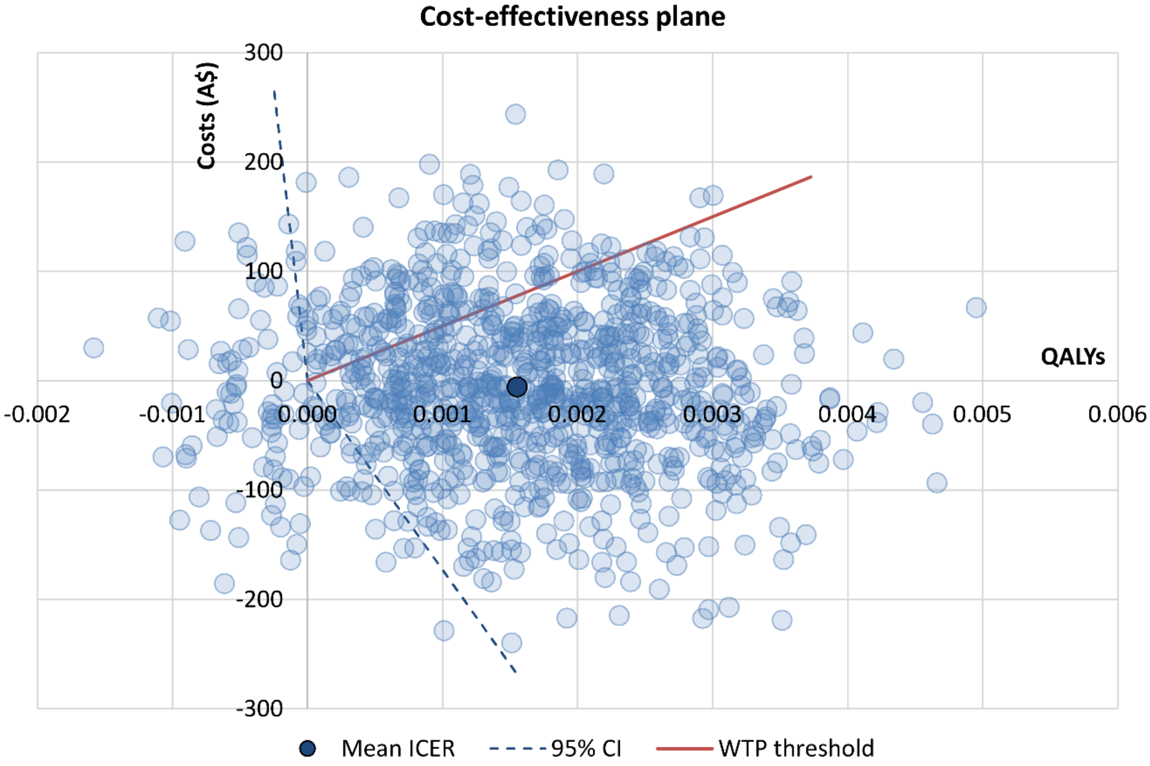

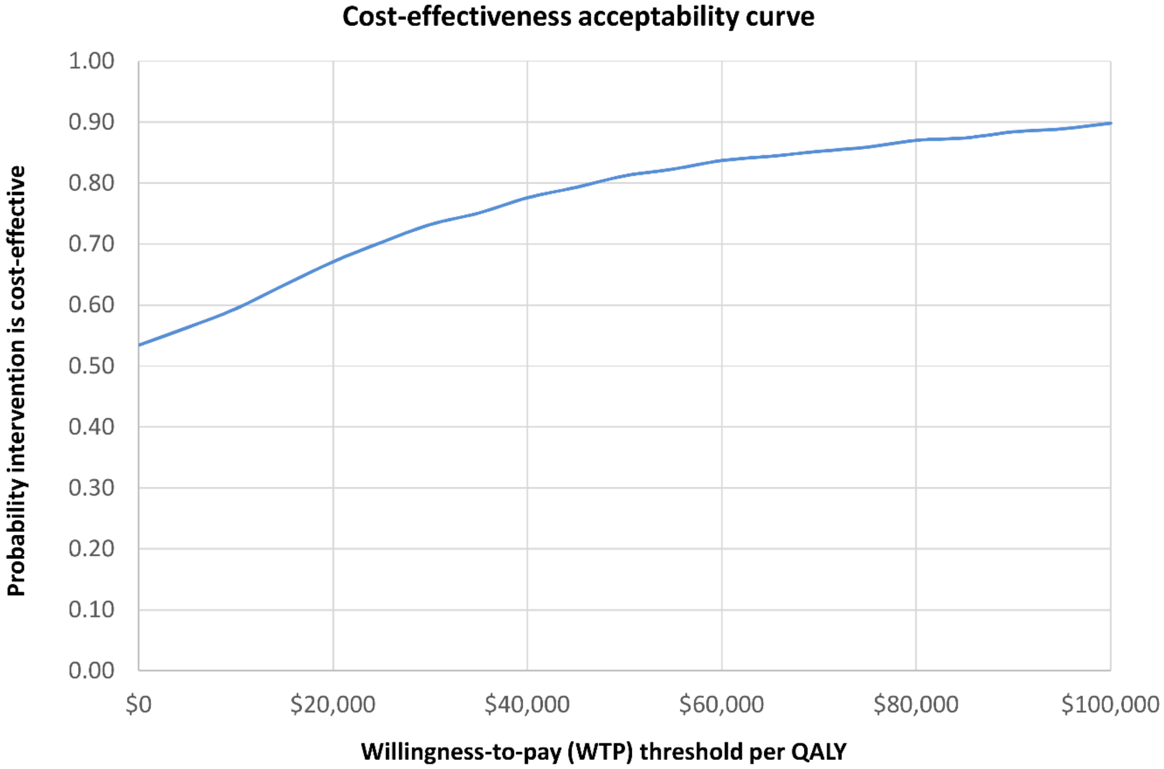

Abbreviations: A$ = Australian dollars; CI = confidence interval; ICER = incremental cost-effectiveness ratio; QALYs = quality-adjusted life years; WTP = willingness-to-pay

Supplementary Figure S7. Cost-effectiveness results for the societal perspective across all participants at 3 months

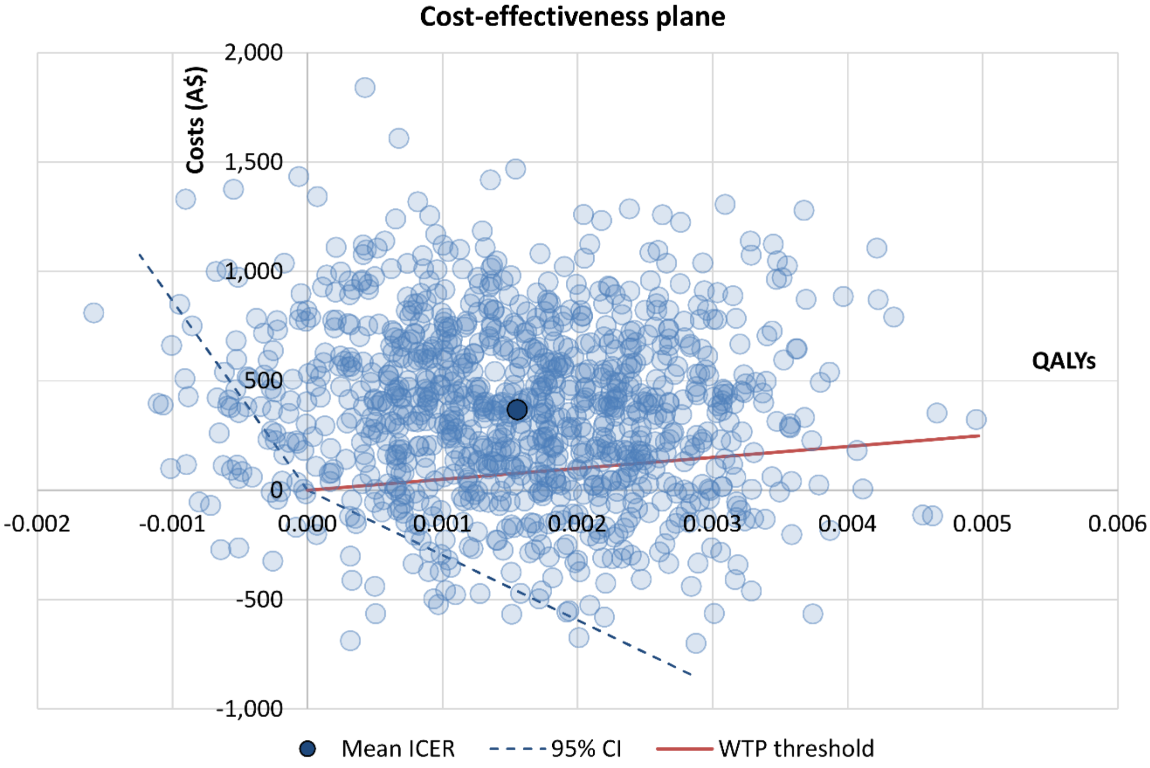

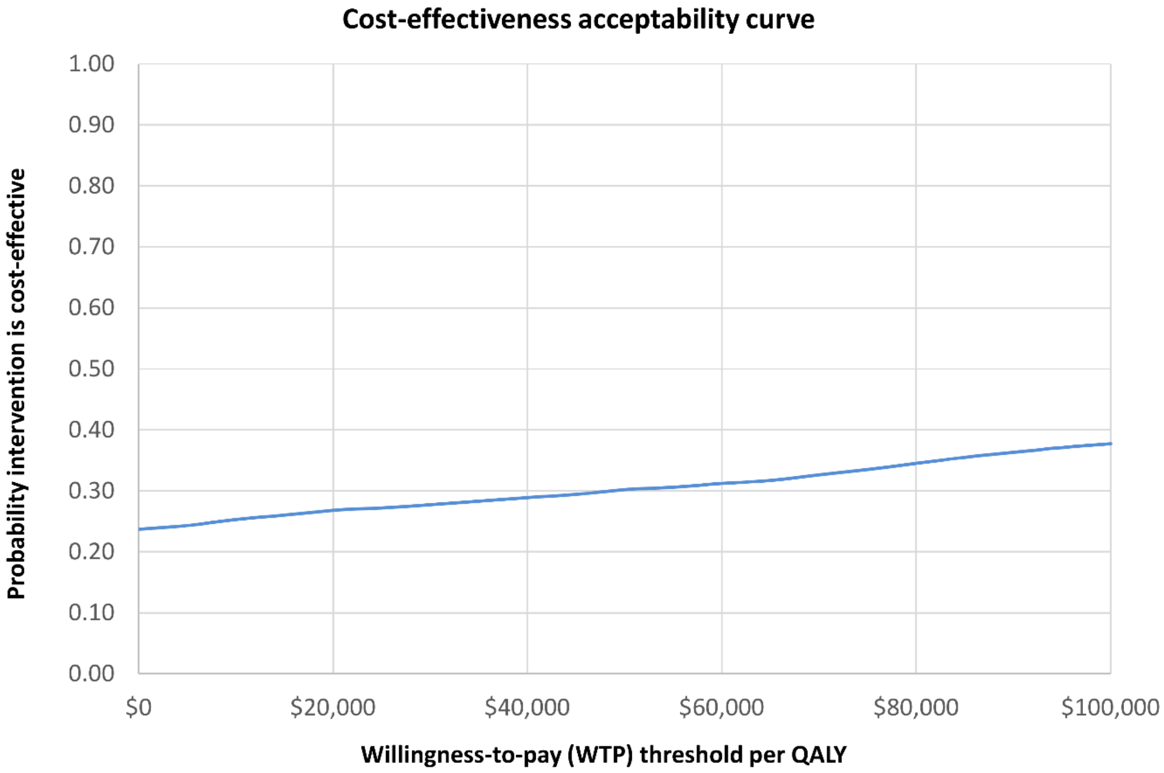

Abbreviations: A$ = Australian dollars; CI = confidence interval; ICER = incremental cost-effectiveness ratio; QALYs = quality-adjusted life years; WTP = willingness to pay

Reference list

1. Miller WR, Rollnick S. Motivational interviewing: Helping people change. 3rd ed. New York: Guildford Press; 2012.
2. Alatawi E, Mendoza A, Miller T. Psychologically-driven requirements engineering: a case study in depression care. 25th Australasian Software Engineering Conference (ASWEC). Adelaide; 2018.
3. Wachtler C, Coe A, Davidson S, et al. Development of a Mobile Clinical Prediction Tool to Estimate Future Depression Severity and Guide Treatment in Primary Care: User-Centered Design. JMIR Mhealth Uhealth 2018;6(4):e95.
4. Proudfoot J, Goldberg D, Mann A, et al. Computerized, interactive, multimedia cognitive-behavioural program for anxiety and depression in general practice. Psychol Med 2003;33(2):217-27.
5. Newby JM, Mackenzie A, Williams AD, et al. Internet cognitive behavioural therapy for mixed anxiety and depression: a randomized controlled trial and evidence of effectiveness in primary care. Psychol Med 2013;43(12):2635-48.
6. Archer J, Bower P, Gilbody S, et al. Collaborative care for depression and anxiety problems. Cochrane Database Syst Rev 2012;10:CD006525.
7. Bower P, Gilbody S, Richards D, et al. Collaborative care for depression in primary care. Making sense of a complex intervention: systematic review and meta-regression. Br J Psychiatry 2006;189:484-93.
8. Miller CJ, Grogan-Kaylor A, Perron BE, et al. Collaborative chronic care models for mental health conditions: cumulative meta-analysis and metaregression to guide future research and implementation. Med Care 2013;51(10):922-30.
9. Thota AB, Sipe TA, Byard GJ, et al. Collaborative care to improve the management of depressive disorders: a community guide systematic review and meta-analysis. Am J Prev Med 2012;42(5):525-38.
10. Sanders GD, Neumann PJ, Basu A, et al. Recommendations for Conduct, Methodological Practices, and Reporting of Cost-effectiveness Analyses: Second Panel on Cost-Effectiveness in Health and Medicine. JAMA 2016;316(10):1093-103.
11. Solomon D, Proudfoot J, Clarke J, Christensen H. e-CBT (myCompass), Antidepressant Medication, and Face-to-Face Psychological Treatment for Depression in Australia: A Cost-Effectiveness Comparison. J Med Internet Res 2015;17(11):e255.
12. Clinical Research Unit for Anxiety and Depression. THIS WAY UP [Online]. St Vincent's Hospital Sydney. 2020 [cited 5 July 2020]; Available from: https://thiswayup.org.au/
13. Lee YC, Gao L, Dear BF, et al. The cost-effectiveness of the online Mindspot Clinic for the treatment of depression and anxiety in Australia. J Ment Health Policy Econ 2017;20(4):155-66.
14. Services Australia. Medicare Item Reports [Online]. Commonwealth of Australia. 2020 [cited 21 June 2020]; Available from: http://medicarestatistics.humanservices.gov.au/statistics/mbs_item.jsp
15. Australian Bureau of Statistics. 6306.0 - Employee Earnings and Hours, Australia, May 2018 [Online]. Commonwealth of Australia. 2019 [cited 21 June 2020]; Available

from: https://www.abs.gov.au/AUSSTATS/abs@.nsf/Lookup/6306.0Main+Features1May%202018

16. Services Australia. Pharmaceutical Benefits Schedule Item Reports [Online]. Commonwealth of Australia. 2020 [cited 21 June 2020]; Available from: http://medicarestatistics.humanservices.gov.au/statistics/pbs_item.jsp

17. Independent Hospital Pricing Authority. National Hospital Cost Data Collection Report, Public Sector, Round 21 (Financial year 2016-17) [Online]. IHPA. 2020 [cited 21 June 2020]; Available from: https://www.ihpa.gov.au/publications/national-hospital-cost-data-collection-report-public-sector-round-21-financial-year

18. Australian Institute of Health and Welfare. Health expenditure Australia 2017-18. Health and welfare expenditure series no. 65. Cat. no. HWE 77. Canberra: AIHW; 2019.

19. Jacobs P, Fassbender K. The measurement of indirect costs in the health economics evaluation literature. A review. Int J Technol Assess Health Care 1998;14(4):799-808.
