# Supplementary material for: Economic evaluation of the Target-D platform to match depression management to severity prognosis in primary care: A within-trial cost-utility analysis
Source: PLoS One. 2022 May 25;17(5):e0268948. doi: 10.1371/journal.pone.0268948 (PMC9132336; doi:10.1371/journal.pone.0268948)
Supplement: S2 Appendix — (PDF) [file pone.0268948.s002.pdf]

```
  ___  ____  ____  ____  ____(R)
 /__    /   ____/   /   ____/
___/   /   /___/   /   /___/
  Statistics/Data analysis

     User: Yong Yi Lee
  Project: Target_D
```

```
1 . codebook
```

NOTE: Follow-up periods are denoted by the following suffixes appended to each variable name.

_t = Baseline
_0 = Baseline
_1 = Baseline
_2 = 3-month follow-up
_3 = 12-month follow-up

---

study_id — **Study identifier**

```
                  Type: Numeric (int)

                 Range: [1,4371]                     Units: 1
         Unique values: 1,868                    Missing .: 0/1,868

                  Mean: 2403.31
             Std. dev.: 1206.56

           Percentiles:     10%       25%       50%       75%       90%
                            681    1389.5      2445    3445.5      3992
```

---

group — **Randomisation group**

```
                  Type: Numeric (byte)
                 Label: group_lbl

                 Range: [0,1]                        Units: 1
         Unique values: 2                        Missing .: 0/1,868

            Tabulation: Freq.   Numeric  Label
                          935         0  Control
                          933         1  Intervention
```

---

phqdep_total_impute_t — **Depression - PHQ-9 score (baseline)**

```
                  Type: Numeric (float)

                 Range: [0,27]                       Units: 1
         Unique values: 28                       Missing .: 0/1,868

                  Mean: 9.23233
             Std. dev.: 5.71362

           Percentiles:     10%       25%       50%       75%       90%
                              3         5         8        13        17
```

---

clinic_0 — **Clinic**

```
                  Type: Numeric (byte)

                 Range: [1,14]                       Units: 1
         Unique values: 14                       Missing .: 0/1,868

                  Mean: 8.93737
             Std. dev.: 3.65385

           Percentiles:     10%       25%       50%       75%       90%
                              4         5        10        12        13
```

---

risk — **Prognostic group**

```
                  Type: Numeric (byte)
                 Label: risk

                 Range: [1,3]                        Units: 1
         Unique values: 3                        Missing .: 0/1,868
```

```
      Tabulation: Freq.   Numeric  Label
                  1,357         1  Mild
                    288         2  Moderate
                    223         3  Severe
```

---

age_num_0 — **Age**

```
            Type: Numeric (double)

           Range: [18,65]                     Units: 1
   Unique values: 48                      Missing .: 0/1,868

            Mean:  35.531
       Std. dev.: 12.0869

     Percentiles:     10%       25%       50%       75%       90%
                       22        26        33        44        55
```

---

gender_0 — **Gender**

```
            Type: Numeric (byte)
           Label: gender

           Range: [0,2]                       Units: 1
   Unique values: 3                       Missing .: 0/1,868

      Tabulation: Freq.   Numeric  Label
                    590         0  Male
                  1,270         1  Female
                      8         2  Other
```

---

costintv — **Intervention cost (applies to intervention group only)**

```
            Type: Numeric (float)

           Range: [0,676.96]                  Units: .01
   Unique values: 4                       Missing .: 0/1,868

      Tabulation: Freq.  Value
                    935  0
                    679  14.96
                    143  132.96001
                    111  676.96002
```

SA1_costintv — **Intervention cost under sensitivity analysis incorporating sunk costs for Target-D CPT research & development**

```
            Type: Numeric (float)

           Range: [0,678.3]                   Units: .1
   Unique values: 4                       Missing .: 0/1,868

      Tabulation: Freq.  Value
                    935  0
                    679  16.299999
                    143  134.3
                    111  678.29999
```

SA2_costintv — **Intervention cost under sensitivity analysis with higher unit cost for moderate prognostic group**

```
            Type: Numeric (float)
```

```
          Range: [0,676.96]                 Units: .01
  Unique values: 4                      Missing .: 0/1,868

     Tabulation: Freq.  Value
                   935  0
                   679  14.96
                   143  222.96001
                   111  676.96002
```

---

costtothealthprof_2 — **Total cost of health professional visits (3 months)**

```
           Type: Numeric (float)

          Range: [0,4021.0701]              Units: 1.000e-06
  Unique values: 711                    Missing .: 809/1,868

           Mean: 340.673
      Std. dev.: 418.347

    Percentiles:     10%       25%       50%       75%       90%
                       0   42.5987   228.087   491.278   806.205
```

---

costtotervisit_2 — **Total cost of emergency department visits (3 months)**

```
           Type: Numeric (float)

          Range: [0,2236.36]                Units: .01
  Unique values: 8                      Missing .: 846/1,868

     Tabulation: Freq.  Value
                   953  0
                     1  4.5
                     2  554.59003
                    53  559.09003
                     6  659.09003
                     5  1118.1801
                     1  1188.1801
                     1  2236.3601
                   846  .
```

---

costtotovnstay_2 — **Total cost of overnight hospital stays (3 months)**

```
           Type: Numeric (float)

          Range: [0,26772.9]                Units: .1
  Unique values: 11                     Missing .: 847/1,868

           Mean: 252.687
      Std. dev.:  1899.1

    Percentiles:     10%       25%       50%       75%       90%
                       0         0         0         0         0
```

---

costtotdiagtest_2 — **Total cost of diagnostic tests (3 months)**

```
           Type: Numeric (float)

          Range: [0,2028.84]                Units: .01
  Unique values: 130                    Missing .: 835/1,868

           Mean: 67.4869
      Std. dev.:  165.32
```

Percentiles:	10%	25%	50%	75%	90%
	**0**	**0**	**0**	**49.76**	**177.23**

---

costtotmed_2 — **Total cost of medications (3 months)**

Type: Numeric (**float**)

Range: [**0**,**488.25**]   Units: **.01**
Unique values: **118**   Missing .: **844/1,868**

Mean: **17.6252**
Std. dev.: **41.426**

Percentiles:	10%	25%	50%	75%	90%
	**0**	**0**	**0**	**23.17**	**55.93**

---

costtothealthprof_3 — **Total cost of health professional visits (12 months)**

Type: Numeric (**float**)

Range: [**0**,**6171.1445**]   Units: **1.000e-06**
Unique values: **602**   Missing .: **1,070/1,868**

Mean: **503.983**
Std. dev.: **586.04**

Percentiles:	10%	25%	50%	75%	90%
	**0**	**83.852**	**301.507**	**741.049**	**1280.5**

---

costtotervisit_3 — **Total cost of emergency department visits (12 months)**

Type: Numeric (**float**)

Range: [**0**,**1677.27**]   Units: **.01**
Unique values: **9**   Missing .: **1,113/1,868**

Tabulation:

Freq.	Value
**687**	**0**
**1**	**99**
**4**	**554.59003**
**55**	**559.09003**
**1**	**563.59003**
**2**	**659.09003**
**3**	**1118.1801**
**1**	**1218.1801**
**1**	**1677.27**
**1,113**	**.**

---

costtotovnstay_3 — **Total cost of overnight hospital stays (12 months)**

Type: Numeric (**float**)

Range: [**0**,**24339**]   Units: **.1**
Unique values: **10**   Missing .: **1,113/1,868**

Mean: **538.359**
Std. dev.: **2365.24**

Percentiles:	10%	25%	50%	75%	90%
	**0**	**0**	**0**	**0**	**0**

---

costtotdiagtest_3 — **Total cost of diagnostic tests (12 months)**

Type: Numeric (**float**)

Range: [**0**,**2419.07**] Units: **.01**
Unique values: **145** Missing .: **1,104**/**1,868**

Mean: **101.61**
Std. dev.: **217.935**

Percentiles:	10%	25%	50%	75%	90%
	**0**	**0**	**24.88**	**82.16**	**279.91**

---

costtotmed_3 — **Total cost of medications (12 months)**

Type: Numeric (**float**)

Range: [**0**,**995.05**] Units: **.01**
Unique values: **114** Missing .: **1,107**/**1,868**

Mean: **53.5464**
Std. dev.: **115.896**

Percentiles:	10%	25%	50%	75%	90%
	**0**	**0**	**0**	**79.02**	**153.03**

---

costtotprodloss_2 — **Total productivity losses (3 months)**

Type: Numeric (**float**)

Range: [**0**,**45090.672**] Units: **.0001**
Unique values: **238** Missing .: **841**/**1,868**

Mean: **4697.63**
Std. dev.: **5586.42**

Percentiles:	10%	25%	50%	75%	90%
	**0**	**0**	**3119.77**	**7737.8**	**11561.3**

---

costtotprodloss_3 — **Total productivity losses (12 months)**

Type: Numeric (**float**)

Range: [**0**,**82854.328**] Units: **.0001**
Unique values: **253** Missing .: **1,103**/**1,868**

Mean: **11459**
Std. dev.: **12577.1**

Percentiles:	10%	25%	50%	75%	90%
	**0**	**0**	**8438.29**	**18381.3**	**26852**

AQoL8DUtility_1 — **AQoL-8D utility score (baseline)**

Type: Numeric (**float**)

Range: [**.11649436**,**1**] Units: **1.000e-08**
Unique values: **1,684** Missing .: **184**/**1,868**

Mean: **.569382**
Std. dev.: **.196468**

Percentiles:	10%	25%	50%	75%	90%
	**.3115**	**.422853**	**.553744**	**.714437**	**.847783**

AQoL8DUtility_2 — **AQoL-8D utility score (3 months)**

Type: Numeric (**float**)

Range: [**.11332551**,**1**] Units: **1.000e-08**
Unique values: **1,012** Missing .: **856**/**1,868**

Mean: **.592451**
Std. dev.: **.203951**

Percentiles:	10%	25%	50%	75%	90%
	**.321678**	**.431112**	**.58164**	**.749469**	**.878988**

AQoL8DUtility_3 — **AQoL-8D utility score (12 months)**

Type: Numeric (**float**)

Range: [**.14523593**,**.99968475**] Units: **1.000e-08**
Unique values: **750** Missing .: **1,118**/**1,868**

Mean: **.609107**
Std. dev.: **.204689**

Percentiles:	10%	25%	50%	75%	90%
	**.330536**	**.45506**	**.607112**	**.782252**	**.888144**

---

phqdep_total_impute_2 — **Depression - PHQ-9 score (3 months)**

Type: Numeric (**float**)

Range: [**0**,**27**] Units: **1**
Unique values: **28** Missing .: **606**/**1,868**

Mean: **8.73296**
Std. dev.: **6.2971**

Percentiles:	10%	25%	50%	75%	90%
	**1**	**4**	**7**	**13**	**18**

---

phqdep_total_impute_3 — **Depression - PHQ-9 score (12 months)**

Type: Numeric (**float**)

Range: [**0**,**27**] Units: **1**
Unique values: **28** Missing .: **703**/**1,868**

Mean: **8.11674**
Std. dev.: **6.03413**

Percentiles:	10%	25%	50%	75%	90%
	**1**	**3**	**7**	**12**	**17**

---

education_rec_0 — **Highest level of education attained**

Type: Numeric (**float**)
Label: **education_rec**

Range: [**1**,**4**]   Units: **1**
Unique values: **4**   Missing .: **0**/**1,868**

Tabulation:

Freq.	Numeric	Label
**221**	**1**	<Year 10/Year 10/equiv/Year 11/equiv
**282**	**2**	Year 12/equiv
**441**	**3**	Certificate/diploma
**924**	**4**	Bachelor degree or higher

---

employment_0 — **Employment status**

Type: Numeric (**byte**)
Label: **employment**

Range: [**0**,**3**]   Units: **1**
Unique values: **3**   Missing .: **0**/**1,868**

Tabulation:

Freq.	Numeric	Label
**1,353**	**0**	Employed/working
**211**	**2**	Unemployed and looking for work
**304**	**3**	Neither working nor looking for work

---

card_none_0 — **Health care card status**

Type: Numeric (**float**)
Label: **yesno**

Range: [**0**,**1**]   Units: **1**
Unique values: **2**   Missing .: **0**/**1,868**

Tabulation:

Freq.	Numeric	Label
**473**	**0**	No
**1,395**	**1**	Yes

---

illness_t — **Long-term illness status**

Type: Numeric (**byte**)
Label: **yesno**

Range: [**0**,**1**]   Units: **1**
Unique values: **2**   Missing .: **0**/**1,868**

Tabulation:

Freq.	Numeric	Label
**1,353**	**0**	No
**515**	**1**	Yes

---

health_0 — **Self-rated health**

Type: Numeric (**byte**)
Label: **health**

Range: [**1**,**5**]   Units: **1**
Unique values: **5**   Missing .: **0**/**1,868**

Tabulation:

Freq.	Numeric	Label
**102**	**1**	Excellent
**580**	**2**	Very good
**779**	**3**	Good
**319**	**4**	Fair
**88**	**5**	Poor

live_alone_0 — **Live alone?**

Type: Numeric (**float**)
Label: **yesno**

Range: [**0**,**1**]   Units: **1**
Unique values: **2**   Missing .: **0/1,868**

Tabulation:

Freq.	Numeric	Label
**1,629**	**0**	No
**239**	**1**	Yes

incomerec2_t — **Ability to manage on available income**

Type: Numeric (**float**)
Label: **incomerec**

Range: [**1**,**2**]   Units: **1**
Unique values: **2**   Missing .: **0/1,868**

Tabulation:

Freq.	Numeric	Label
**1,649**	**1**	Easily/Not too bad
**219**	**2**	Difficult some of the time

psychpast_visited_0 — **Visited psychologist or counsellor in 12 months**

Type: Numeric (**float**)
Label: **yesno**

Range: [**0**,**1**]   Units: **1**
Unique values: **2**   Missing .: **0/1,868**

Tabulation:

Freq.	Numeric	Label
**1,078**	**0**	No
**790**	**1**	Yes

antidepressants_0 — **Currently using antidepressants?**

Type: Numeric (**float**)
Label: **yesno**

Range: [**0**,**1**]   Units: **1**
Unique values: **2**   Missing .: **0/1,868**

Tabulation:

Freq.	Numeric	Label
**1,452**	**0**	No
**416**	**1**	Yes

```


*************************************************************************
*************************************************************************
***********                                                   ***********
***********         STATA DO-FILE FOR TARGET-D ANALYSIS       ***********
***********                                                   ***********
*************************************************************************
*************************************************************************


* NOTE: Use code below to conduct analysis of Target-D data set
*
* Code is split into 3 modules:
* (1) Complete case analysis;
* (2) Multiple imputation; and
* (3) Bootstrap analysis.
*
*



******************
***************   MODULE 1 - COMPLETE CASE ANALYSIS
************

****************************
***   DATA PREPARATION   ***
****************************

*** PREPARATORY CODE FOR COMPLETE CASE ANALYSIS

** Set working directory

cd "C:\Stata"


** Load original dataset, keep in-scope variables and save the revised dataset

clear all
use "C:\Stata\TargetD_MERGED_edited.dta", replace

global var_sid study_id

global var_reg group phqdep_total_impute_t clinic_0 risk /// *Regular_variables
               age_num_0 gender_0 costintv SA1_costintv SA2_costintv

global var_imp costtothealthprof_2 costtotervisit_2 /// *Imputed_variables
               costtotovnstay_2 costtotdiagtest_2 costtotmed_2 ///
               costtothealthprof_3 costtotervisit_3 costtotovnstay_3 ///
               costtotdiagtest_3 costtotmed_3 ///
               costtotprodloss_2 costtotprodloss_3 ///
               AQoL8DUtility_1 AQoL8DUtility_2 AQoL8DUtility_3 ///
               phqdep_total_impute_2 phqdep_total_impute_3

global var_adj education_rec_0 employment_0  /// *SA3_adjustment_variables
               card_none_0 illness_t  health_0  live_alone_0 ///
               incomerec2_t psychpast_visited_0  antidepressants_0

keep $var_sid $var_reg $var_imp $var_adj
order $var_sid $var_reg $var_imp $var_adj

save "C:\Stata\TargetD_MERGED_inscope.dta", replace


** Load revised dataset containing only the in-scope variables

clear all
use "C:\Stata\TargetD_MERGED_inscope.dta", replace


** Add aggregate cost variables by study perspective

* Health sector perspective

gen costhealthsector_2 = costtothealthprof_2 + costtotervisit_2 + ///
                         costtotovnstay_2 + costtotdiagtest_2 + ///
                         costtotmed_2 + costintv

```

```
gen costhealthsector_3 = costtothealthprof_3 + costtotervisit_3 + ///
                         costtotovnstay_3 + costtotdiagtest_3 + ///
                         costtotmed_3

gen costhealthsectortot = costhealthsector_2 + costhealthsector_3

* Societal perspective

gen costsocietal_2 = costhealthsector_2 + costtotprodloss_2

gen costsocietal_3 = costhealthsector_3 + costtotprodloss_3

gen costsocietaltot = costsocietal_2 + costsocietal_3


** Add aggregate QALY variables

gen qaly_2 = 0.5*(AQoL8DUtility_1 + AQoL8DUtility_2)*(3/12)
gen qaly_3 = 0.5*(AQoL8DUtility_2 + AQoL8DUtility_3)*(9/12)
gen qalytot = qaly_2 + qaly_3



*********************************************
***   TARGET_D - COMPLETE CASE ANALYSIS   ***
*********************************************

*** GLM ANALYSIS OF COST VARIABLES AT 3-MONTH FOLLOW-UP (T2)

** Analysis of health sector costs - 3-month follow-up (T2)

* Adjusted for baseline outcome and stratification factors

glm costhealthsector_2 i.group phqdep_total_impute_t i.clinic_0 i.risk, family(gamma) link(log) eform

* Subgroup analysis

glm costhealthsector_2 i.group phqdep_total_impute_t i.clinic_0 if risk==1, family(gamma) link(log) eform
glm costhealthsector_2 i.group phqdep_total_impute_t i.clinic_0 if risk==2, family(gamma) link(log) eform
glm costhealthsector_2 i.group phqdep_total_impute_t i.clinic_0 if risk==3, family(gamma) link(log) eform


** Analysis of societal costs - 3-month follow-up (T2)

* Adjusted for baseline outcome and stratification factors

glm costsocietal_2 i.group phqdep_total_impute_t i.clinic_0 i.risk, family(gamma) link(log) eform

* Subgroup analysis

glm costsocietal_2 i.group phqdep_total_impute_t i.clinic_0 if risk==1, family(gamma) link(log) eform
glm costsocietal_2 i.group phqdep_total_impute_t i.clinic_0 if risk==2, family(gamma) link(log) eform
glm costsocietal_2 i.group phqdep_total_impute_t i.clinic_0 if risk==3, family(gamma) link(log) eform



*** GLM ANALYSIS OF COST VARIABLES AT 12-MONTH FOLLOW-UP (T2+T3)

** Analysis of health sector costs - 12-month follow-up (T2+T3)

* Adjusted for baseline outcome and stratification factors

glm costhealthsectortot i.group phqdep_total_impute_t i.clinic_0 i.risk, family(gamma) link(log) eform

* Subgroup analysis

glm costhealthsectortot i.group phqdep_total_impute_t i.clinic_0 if risk==1, family(gamma) link(log) eform
glm costhealthsectortot i.group phqdep_total_impute_t i.clinic_0 if risk==2, family(gamma) link(log) eform
```

```
glm costhealthsectortot i.group phqdep_total_impute_t i.clinic_0 if risk==3, family(gamma) link(log) eform


** Analysis of societal costs - 12-month follow-up (T2+T3)

* Adjusted for baseline outcome and stratification factors

glm costsocietaltot i.group phqdep_total_impute_t i.clinic_0 i.risk, family(gamma) link(log) eform

* Subgroup analysis

glm costsocietaltot i.group phqdep_total_impute_t i.clinic_0 if risk==1, family(gamma) link(log) eform
glm costsocietaltot i.group phqdep_total_impute_t i.clinic_0 if risk==2, family(gamma) link(log) eform
glm costsocietaltot i.group phqdep_total_impute_t i.clinic_0 if risk==3, family(gamma) link(log) eform



*** GLM ANALYSIS OF QALYS AT 3-MONTH FOLLOW-UP (T2)

** Analysis of QALYs - 3-month follow-up (T2)

* Adjusted for baseline utility, baseline outcome and stratification factors

glm qaly_2 i.group AQoL8DUtility_1 phqdep_total_impute_t i.clinic_0 i.risk, family(gaussian) link(identity)

* Subgroup analysis

glm qaly_2 i.group AQoL8DUtility_1 phqdep_total_impute_t i.clinic_0 if risk==1, family(gaussian) link(identity)
glm qaly_2 i.group AQoL8DUtility_1 phqdep_total_impute_t i.clinic_0 if risk==2, family(gaussian) link(identity)
glm qaly_2 i.group AQoL8DUtility_1 phqdep_total_impute_t i.clinic_0 if risk==3, family(gaussian) link(identity)



*** GLM ANALYSIS OF QALYS AT 12-MONTH FOLLOW-UP (T2+T3)

** Analysis of QALYs - 12-month follow-up (T2+T3)

* Adjusted for baseline utility, baseline outcome and stratification factors

glm qalytot i.group AQoL8DUtility_1 phqdep_total_impute_t i.clinic_0 i.risk, family(gaussian) link(identity)

* Subgroup analysis

glm qalytot i.group AQoL8DUtility_1 phqdep_total_impute_t i.clinic_0 if risk==1, family(gaussian) link(identity)
glm qalytot i.group AQoL8DUtility_1 phqdep_total_impute_t i.clinic_0 if risk==2, family(gaussian) link(identity)
glm qalytot i.group AQoL8DUtility_1 phqdep_total_impute_t i.clinic_0 if risk==3, family(gaussian) link(identity)



******************
***************   END OF MODULE 1 - COMPLETE CASE ANALYSIS
************







******************
***************   START OF MODULE 2 - IMPUTATION ANALYSIS
************


****************************
***   DATA PREPARATION   ***
****************************
```

```
*** PREPARATORY CODE FOR MULTIPLE IMPUTATION ANALYSIS

** Set working directory

cd "C:\Stata"


** Load original dataset, keep in-scope variables and save the revised dataset

clear all
use "C:\Stata\TargetD_MERGED_edited.dta", replace

global var_sid study_id

global var_reg group phqdep_total_impute_t clinic_0 risk /// *Regular_variables
               age_num_0 gender_0 costintv SA1_costintv SA2_costintv

global var_imp costtothealthprof_2 costtotervisit_2 /// *Imputed_variables
               costtotovnstay_2 costtotdiagtest_2 costtotmed_2 ///
               costtothealthprof_3 costtotervisit_3 costtotovnstay_3 ///
               costtotdiagtest_3 costtotmed_3 ///
               costtotprodloss_2 costtotprodloss_3 ///
               AQoL8DUtility_1 AQoL8DUtility_2 AQoL8DUtility_3 ///
               phqdep_total_impute_2 phqdep_total_impute_3

global var_adj education_rec_0 employment_0  /// *SA3_adjustment_variables
               card_none_0 illness_t  health_0  live_alone_0 ///
               incomerec2_t psychpast_visited_0  antidepressants_0

keep $var_sid $var_reg $var_imp $var_adj
order $var_sid $var_reg $var_imp $var_adj

save "C:\Stata\TargetD_MERGED_inscope.dta", replace


** Load revised dataset containing only the in-scope variables

clear all
use "C:\Stata\TargetD_MERGED_inscope.dta", replace



*** SET UP IMPUTATION DATA SET AND REGISTER VARIABLES

** Duplicate existing variables for the multiple imputation analysis

gen costtothealthprof_2_imp = costtothealthprof_2
gen costtotervisit_2_imp = costtotervisit_2
gen costtotovnstay_2_imp = costtotovnstay_2
gen costtotdiagtest_2_imp = costtotdiagtest_2
gen costtotmed_2_imp = costtotmed_2

gen costtothealthprof_3_imp = costtothealthprof_3
gen costtotervisit_3_imp = costtotervisit_3
gen costtotovnstay_3_imp = costtotovnstay_3
gen costtotdiagtest_3_imp = costtotdiagtest_3
gen costtotmed_3_imp = costtotmed_3

gen costtotprodloss_2_imp = costtotprodloss_2
gen costtotprodloss_3_imp = costtotprodloss_3

gen AQoL8DUtility_1_imp = AQoL8DUtility_1
gen AQoL8DUtility_2_imp = AQoL8DUtility_2
gen AQoL8DUtility_3_imp = AQoL8DUtility_3

gen phqdep_total_impute_2_imp = phqdep_total_impute_2
gen phqdep_total_impute_3_imp = phqdep_total_impute_3


** MI estimation commands

* Declare MI data set

mi set flong

* Register variables to be imputed

mi register imputed costtothealthprof_2_imp costtotervisit_2_imp costtotovnstay_2_imp ///
```

```
                    costtotdiagtest_2_imp costtotmed_2_imp costtothealthprof_3_imp ///
                    costtotervisit_3_imp costtotovnstay_3_imp costtotdiagtest_3_imp ///
                    costtotmed_3_imp costtotprodloss_2_imp costtotprodloss_3_imp ///
                    AQoL8DUtility_1_imp AQoL8DUtility_2_imp AQoL8DUtility_3_imp ///
                    phqdep_total_impute_2_imp phqdep_total_impute_3_imp

mi register regular $var_reg $var_adj

* Perform multiple imputation

mi impute chained ///
    (pmm, knn(10)) AQoL8DUtility_1_imp AQoL8DUtility_2_imp AQoL8DUtility_3_imp ///
                   phqdep_total_impute_2_imp phqdep_total_impute_3_imp ///
                   costtothealthprof_2_imp costtotervisit_2_imp costtotovnstay_2_imp ///
                   costtotdiagtest_2_imp costtotmed_2_imp costtothealthprof_3_imp ///
                   costtotervisit_3_imp costtotovnstay_3_imp costtotdiagtest_3_imp ///
                   costtotmed_3_imp costtotprodloss_2_imp costtotprodloss_3_imp ///
    = i.group i.gender_0 i.clinic_0 age_num_0 education_rec_0 psychpast_visited_0, ///
    by(risk) add(100) burnin(100) rseed(2812281) dots

* Generate (and register) passive variables

mi passive: gen costhealthsector_2_imp = costtothealthprof_2_imp + costtotervisit_2_imp +
///
                                         costtotovnstay_2_imp + costtotdiagtest_2_imp + ///
                                         costtotmed_2_imp + costintv
mi passive: gen costhealthsector_3_imp = costtothealthprof_3_imp + costtotervisit_3_imp +
///
                                         costtotovnstay_3_imp + costtotdiagtest_3_imp + ///
                                         costtotmed_3_imp
mi passive: gen costhealthsectortot_imp = costhealthsector_2_imp + costhealthsector_3_imp

mi passive: gen costsocietal_2_imp = costhealthsector_2_imp + costtotprodloss_2_imp
mi passive: gen costsocietal_3_imp = costhealthsector_3_imp + costtotprodloss_3_imp
mi passive: gen costsocietaltot_imp = costsocietal_2_imp + costsocietal_3_imp

mi passive: gen qaly_2_imp = 0.5*(AQoL8DUtility_1_imp + AQoL8DUtility_2_imp)*(3/12)
mi passive: gen qaly_3_imp = 0.5*(AQoL8DUtility_2_imp + AQoL8DUtility_3_imp)*(9/12)
mi passive: gen qalytot_imp = qaly_2_imp + qaly_3_imp

mi passive: gen SA1_costhealthsector_2_imp = costtothealthprof_2_imp +
costtotervisit_2_imp + ///
                                             costtotovnstay_2_imp + costtotdiagtest_2_imp
+ ///
                                             costtotmed_2_imp + SA1_costintv
mi passive: gen SA1_costhealthsectortot_imp = SA1_costhealthsector_2_imp +
costhealthsector_3_imp

mi passive: gen SA1_costsocietal_2_imp = SA1_costhealthsector_2_imp + costtotprodloss_2_imp
mi passive: gen SA1_costsocietaltot_imp = SA1_costsocietal_2_imp + costsocietal_3_imp

mi passive: gen SA2_costhealthsector_2_imp = costtothealthprof_2_imp +
costtotervisit_2_imp + ///
                                             costtotovnstay_2_imp + costtotdiagtest_2_imp
+ ///
                                             costtotmed_2_imp + SA2_costintv
mi passive: gen SA2_costhealthsectortot_imp = SA2_costhealthsector_2_imp +
costhealthsector_3_imp

mi passive: gen SA2_costsocietal_2_imp = SA2_costhealthsector_2_imp + costtotprodloss_2_imp
mi passive: gen SA2_costsocietaltot_imp = SA2_costsocietal_2_imp + costsocietal_3_imp

* Save copy of the multiple imputation data set

describe, short
save "C:\Stata\TargetD_MERGED_MI_update.dta", replace

* Clear data set from memory

clear all



***********************************************
***   TARGET_D - ANALYSIS OF IMPUTED DATA   ***
***********************************************

*** Set working directory

```

```
cd "C:\Stata"


*** LOAD IMPUTED DATA SET

use "C:\Stata\TargetD_MERGED_MI_update.dta", clear
describe, short


*** BASE CASE STATISTICAL ANALYSES USING IMPUTED DATA SET

** GLM analysis of cost variables

* Analysis of health sector costs at 3-month follow-up (T2)

mi estimate, eform post: glm costhealthsector_2_imp i.group phqdep_total_impute_t i.
clinic_0 i.risk, family(gamma) link(log)
mimrgns group, predict(xb) eform

* Subgroup analysis

mi estimate, eform post: glm costhealthsector_2_imp i.group phqdep_total_impute_t i.
clinic_0 if risk==1, family(gamma) link(log)
mimrgns group, predict(xb) eform

mi estimate, eform post: glm costhealthsector_2_imp i.group phqdep_total_impute_t i.
clinic_0 if risk==2, family(gamma) link(log)
mimrgns group, predict(xb) eform

mi estimate, eform post: glm costhealthsector_2_imp i.group phqdep_total_impute_t i.
clinic_0 if risk==3, family(gamma) link(log)
mimrgns group, predict(xb) eform

* Analysis of health sector costs at 12-month follow-up (T2+T3)

mi estimate, eform post: glm costhealthsectortot_imp i.group phqdep_total_impute_t i.
clinic_0 i.risk, family(gamma) link(log)
mimrgns group, predict(xb) eform

* Subgroup analysis

mi estimate, eform post: glm costhealthsectortot_imp i.group phqdep_total_impute_t i.
clinic_0 if risk==1, family(gamma) link(log)
mimrgns group, predict(xb) eform

mi estimate, eform post: glm costhealthsectortot_imp i.group phqdep_total_impute_t i.
clinic_0 if risk==2, family(gamma) link(log)
mimrgns group, predict(xb) eform

mi estimate, eform post: glm costhealthsectortot_imp i.group phqdep_total_impute_t i.
clinic_0 if risk==3, family(gamma) link(log)
mimrgns group, predict(xb) eform

* Analysis of societal costs at 3-month follow-up (T2)

mi estimate, eform post: glm costsocietal_2_imp i.group phqdep_total_impute_t i.clinic_0 i
.risk, family(gamma) link(log)
mimrgns group, predict(xb) eform

* Subgroup analysis

mi estimate, eform post: glm costsocietal_2_imp i.group phqdep_total_impute_t i.clinic_0
if risk==1, family(gamma) link(log)
mimrgns group, predict(xb) eform

mi estimate, eform post: glm costsocietal_2_imp i.group phqdep_total_impute_t i.clinic_0
if risk==2, family(gamma) link(log)
mimrgns group, predict(xb) eform

mi estimate, eform post: glm costsocietal_2_imp i.group phqdep_total_impute_t i.clinic_0
if risk==3, family(gamma) link(log)
mimrgns group, predict(xb) eform

* Analysis of societal costs at 12-month follow-up (T2+T3)

mi estimate, eform post: glm costsocietaltot_imp i.group phqdep_total_impute_t i.clinic_0
i.risk, family(gamma) link(log)
mimrgns group, predict(xb) eform

```

```
* Subgroup analysis

mi estimate, eform post: glm costsocietaltot_imp i.group phqdep_total_impute_t i.clinic_0 if risk==1, family(gamma) link(log)
mimrgns group, predict(xb) eform

mi estimate, eform post: glm costsocietaltot_imp i.group phqdep_total_impute_t i.clinic_0 if risk==2, family(gamma) link(log)
mimrgns group, predict(xb) eform

mi estimate, eform post: glm costsocietaltot_imp i.group phqdep_total_impute_t i.clinic_0 if risk==3, family(gamma) link(log)
mimrgns group, predict(xb) eform


** GLM analysis of QALY variables

* 3-month follow-up (T2)

mi estimate: glm qaly_2_imp i.group AQoL8DUtility_1_imp phqdep_total_impute_t i.clinic_0 i.risk, family(gaussian) link(identity)
mimrgns group, predict(xb)

* Subgroup analysis

mi estimate: glm qaly_2_imp i.group AQoL8DUtility_1_imp phqdep_total_impute_t i.clinic_0 if risk==1, family(gaussian) link(identity)
mimrgns group, predict(xb)

mi estimate: glm qaly_2_imp i.group AQoL8DUtility_1_imp phqdep_total_impute_t i.clinic_0 if risk==2, family(gaussian) link(identity)
mimrgns group, predict(xb)

mi estimate: glm qaly_2_imp i.group AQoL8DUtility_1_imp phqdep_total_impute_t i.clinic_0 if risk==3, family(gaussian) link(identity)
mimrgns group, predict(xb)

* 12-month follow-up (T2+T3)

mi estimate: glm qalytot_imp i.group AQoL8DUtility_1_imp phqdep_total_impute_t i.clinic_0 i.risk, family(gaussian) link(identity)
mimrgns group, predict(xb)

* Subgroup analysis

mi estimate: glm qalytot_imp i.group AQoL8DUtility_1_imp phqdep_total_impute_t i.clinic_0 if risk==1, family(gaussian) link(identity)
mimrgns group, predict(xb)

mi estimate: glm qalytot_imp i.group AQoL8DUtility_1_imp phqdep_total_impute_t i.clinic_0 if risk==2, family(gaussian) link(identity)
mimrgns group, predict(xb)

mi estimate: glm qalytot_imp i.group AQoL8DUtility_1_imp phqdep_total_impute_t i.clinic_0 if risk==3, family(gaussian) link(identity)
mimrgns group, predict(xb)
```

NOTE: Sensitivity analysis SA1 involves the inclusion of sunk costs for the development of the Target-D CPT (cost of screening changed from $0.96 per person to $2.30 per person).

```
*** SENSITIVITY ANALYSES USING IMPUTED DATA SET

** SA1 -- GLM analysis of cost variables

* Analysis of health sector costs at 3-month follow-up (T2)

mi estimate, eform post: glm SA1_costhealthsector_2_imp i.group phqdep_total_impute_t i.clinic_0 i.risk, family(gamma) link(log)

* Subgroup analysis

mi estimate, eform post: glm SA1_costhealthsector_2_imp i.group phqdep_total_impute_t i.clinic_0 if risk==1, family(gamma) link(log)
mi estimate, eform post: glm SA1_costhealthsector_2_imp i.group phqdep_total_impute_t i.clinic_0 if risk==2, family(gamma) link(log)
mi estimate, eform post: glm SA1_costhealthsector_2_imp i.group phqdep_total_impute_t i.clinic_0 if risk==3, family(gamma) link(log)

* Analysis of health sector costs at 12-month follow-up (T2+T3)
```

```
mi estimate, eform post: glm SA1_costhealthsectortot_imp i.group phqdep_total_impute_t i.clinic_0 i.risk, family(gamma) link(log)

* Subgroup analysis

mi estimate, eform post: glm SA1_costhealthsectortot_imp i.group phqdep_total_impute_t i.clinic_0 if risk==1, family(gamma) link(log)
mi estimate, eform post: glm SA1_costhealthsectortot_imp i.group phqdep_total_impute_t i.clinic_0 if risk==2, family(gamma) link(log)
mi estimate, eform post: glm SA1_costhealthsectortot_imp i.group phqdep_total_impute_t i.clinic_0 if risk==3, family(gamma) link(log)

* Analysis of societal costs at 3-month follow-up (T2)

mi estimate, eform post: glm SA1_costsocietal_2_imp i.group phqdep_total_impute_t i.clinic_0 i.risk, family(gamma) link(log)

* Subgroup analysis

mi estimate, eform post: glm SA1_costsocietal_2_imp i.group phqdep_total_impute_t i.clinic_0 if risk==1, family(gamma) link(log)
mi estimate, eform post: glm SA1_costsocietal_2_imp i.group phqdep_total_impute_t i.clinic_0 if risk==2, family(gamma) link(log)
mi estimate, eform post: glm SA1_costsocietal_2_imp i.group phqdep_total_impute_t i.clinic_0 if risk==3, family(gamma) link(log)

* Analysis of societal costs at 12-month follow-up (T2+T3)

mi estimate, eform post: glm SA1_costsocietaltot_imp i.group phqdep_total_impute_t i.clinic_0 i.risk, family(gamma) link(log)

* Subgroup analysis

mi estimate, eform post: glm SA1_costsocietaltot_imp i.group phqdep_total_impute_t i.clinic_0 if risk==1, family(gamma) link(log)
mi estimate, eform post: glm SA1_costsocietaltot_imp i.group phqdep_total_impute_t i.clinic_0 if risk==2, family(gamma) link(log)
mi estimate, eform post: glm SA1_costsocietaltot_imp i.group phqdep_total_impute_t i.clinic_0 if risk==3, family(gamma) link(log)
```

NOTE: Sensitivity analysis SA2 involves a higher unit cost for the clinician-guided iCBT course delivered to the moderate prognostic group (unit cost changed from $132 per person to $222 per person).

```
** SA2 -- GLM analysis of cost variables

* Analysis of health sector costs at 3-month follow-up (T2)

mi estimate, eform post: glm SA2_costhealthsector_2_imp i.group phqdep_total_impute_t i.clinic_0 i.risk, family(gamma) link(log)

* Subgroup analysis

mi estimate, eform post: glm SA2_costhealthsector_2_imp i.group phqdep_total_impute_t i.clinic_0 if risk==1, family(gamma) link(log)
mi estimate, eform post: glm SA2_costhealthsector_2_imp i.group phqdep_total_impute_t i.clinic_0 if risk==2, family(gamma) link(log)
mi estimate, eform post: glm SA2_costhealthsector_2_imp i.group phqdep_total_impute_t i.clinic_0 if risk==3, family(gamma) link(log)

* Analysis of health sector costs at 12-month follow-up (T2+T3)

mi estimate, eform post: glm SA2_costhealthsectortot_imp i.group phqdep_total_impute_t i.clinic_0 i.risk, family(gamma) link(log)

* Subgroup analysis

mi estimate, eform post: glm SA2_costhealthsectortot_imp i.group phqdep_total_impute_t i.clinic_0 if risk==1, family(gamma) link(log)
mi estimate, eform post: glm SA2_costhealthsectortot_imp i.group phqdep_total_impute_t i.clinic_0 if risk==2, family(gamma) link(log)
mi estimate, eform post: glm SA2_costhealthsectortot_imp i.group phqdep_total_impute_t i.clinic_0 if risk==3, family(gamma) link(log)

* Analysis of societal costs at 3-month follow-up (T2)

mi estimate, eform post: glm SA2_costsocietal_2_imp i.group phqdep_total_impute_t i.clinic_0 i.risk, family(gamma) link(log)

* Subgroup analysis

mi estimate, eform post: glm SA2_costsocietal_2_imp i.group phqdep_total_impute_t i.
```

```
clinic_0 if risk==1, family(gamma) link(log)
mi estimate, eform post: glm SA2_costsocietal_2_imp i.group phqdep_total_impute_t i.clinic_0 if risk==2, family(gamma) link(log)
mi estimate, eform post: glm SA2_costsocietal_2_imp i.group phqdep_total_impute_t i.clinic_0 if risk==3, family(gamma) link(log)

* Analysis of societal costs at 12-month follow-up (T2+T3)

mi estimate, eform post: glm SA2_costsocietaltot_imp i.group phqdep_total_impute_t i.clinic_0 i.risk, family(gamma) link(log)

* Subgroup analysis

mi estimate, eform post: glm SA2_costsocietaltot_imp i.group phqdep_total_impute_t i.clinic_0 if risk==1, family(gamma) link(log)
mi estimate, eform post: glm SA2_costsocietaltot_imp i.group phqdep_total_impute_t i.clinic_0 if risk==2, family(gamma) link(log)
mi estimate, eform post: glm SA2_costsocietaltot_imp i.group phqdep_total_impute_t i.clinic_0 if risk==3, family(gamma) link(log)


** SA3 -- GLM analysis of cost and QALY variables after adjustment for variables
**        associated with non-response to the primary outcome measure (PHQ-9 score)

* Analysis of health sector costs at 3-month follow-up (T2)

mi estimate, eform post: glm costhealthsector_2_imp i.group phqdep_total_impute_t i.clinic_0 i.risk age_num_0 i.gender_0 i.education_rec_0 i.employment_0  i.card_none_0 i.illness_t  i.health_0  i.live_alone_0 i.incomerec2_t i.psychpast_visited_0  i.antidepressants_0, family(gamma) link(log)

* Subgroup analysis

mi estimate, eform post: glm costhealthsector_2_imp i.group phqdep_total_impute_t i.clinic_0 age_num_0 i.gender_0 i.education_rec_0 i.employment_0  i.card_none_0 i.illness_t  i.health_0  i.live_alone_0 i.incomerec2_t i.psychpast_visited_0  i.antidepressants_0 if risk==1, family(gamma) link(log)
mi estimate, eform post: glm costhealthsector_2_imp i.group phqdep_total_impute_t i.clinic_0 age_num_0 i.gender_0 i.education_rec_0 i.employment_0  i.card_none_0 i.illness_t  i.health_0  i.live_alone_0 i.incomerec2_t i.psychpast_visited_0  i.antidepressants_0 if risk==2, family(gamma) link(log)
mi estimate, eform post: glm costhealthsector_2_imp i.group phqdep_total_impute_t i.clinic_0 age_num_0 i.gender_0 i.education_rec_0 i.employment_0  i.card_none_0 i.illness_t  i.health_0  i.live_alone_0 i.incomerec2_t i.psychpast_visited_0  i.antidepressants_0 if risk==3, family(gamma) link(log)

* Analysis of health sector costs at 12-month follow-up (T2+T3)

mi estimate, eform post: glm costhealthsectortot_imp i.group phqdep_total_impute_t i.clinic_0 i.risk age_num_0 i.gender_0 i.education_rec_0 i.employment_0  i.card_none_0 i.illness_t  i.health_0  i.live_alone_0 i.incomerec2_t i.psychpast_visited_0  i.antidepressants_0, family(gamma) link(log)

* Subgroup analysis

mi estimate, eform post: glm costhealthsectortot_imp i.group phqdep_total_impute_t i.clinic_0 age_num_0 i.gender_0 i.education_rec_0 i.employment_0  i.card_none_0 i.illness_t  i.health_0  i.live_alone_0 i.incomerec2_t i.psychpast_visited_0  i.antidepressants_0 if risk==1, family(gamma) link(log)
mi estimate, eform post: glm costhealthsectortot_imp i.group phqdep_total_impute_t i.clinic_0 age_num_0 i.gender_0 i.education_rec_0 i.employment_0  i.card_none_0 i.illness_t  i.health_0  i.live_alone_0 i.incomerec2_t i.psychpast_visited_0  i.antidepressants_0 if risk==2, family(gamma) link(log)
mi estimate, eform post: glm costhealthsectortot_imp i.group phqdep_total_impute_t i.clinic_0 age_num_0 i.gender_0 i.education_rec_0 i.employment_0  i.card_none_0 i.illness_t  i.health_0  i.live_alone_0 i.incomerec2_t i.psychpast_visited_0  i.antidepressants_0 if risk==3, family(gamma) link(log)

* Analysis of societal costs at 3-month follow-up (T2)

mi estimate, eform post: glm costsocietal_2_imp i.group phqdep_total_impute_t i.clinic_0 i.risk age_num_0 i.gender_0 i.education_rec_0 i.employment_0  i.card_none_0 i.illness_t  i.health_0  i.live_alone_0 i.incomerec2_t i.psychpast_visited_0  i.antidepressants_0, family(gamma) link(log)

* Subgroup analysis

mi estimate, eform post: glm costsocietal_2_imp i.group phqdep_total_impute_t i.clinic_0 age_num_0 i.gender_0 i.education_rec_0 i.employment_0  i.card_none_0 i.illness_t  i.
```

```
health_0  i.live_alone_0 i.incomerec2_t i.psychpast_visited_0  i.antidepressants_0 if risk ==1, family(gamma) link(log)
mi estimate, eform post: glm costsocietal_2_imp i.group phqdep_total_impute_t i.clinic_0 age_num_0 i.gender_0 i.education_rec_0 i.employment_0  i.card_none_0 i.illness_t  i.health_0  i.live_alone_0 i.incomerec2_t i.psychpast_visited_0  i.antidepressants_0 if risk ==2, family(gamma) link(log)
mi estimate, eform post: glm costsocietal_2_imp i.group phqdep_total_impute_t i.clinic_0 age_num_0 i.gender_0 i.education_rec_0 i.employment_0  i.card_none_0 i.illness_t  i.health_0  i.live_alone_0 i.incomerec2_t i.psychpast_visited_0  i.antidepressants_0 if risk ==3, family(gamma) link(log)

* Analysis of societal costs at 12-month follow-up (T2+T3)

mi estimate, eform post: glm costsocietaltot_imp i.group phqdep_total_impute_t i.clinic_0 i.risk age_num_0 i.gender_0 i.education_rec_0 i.employment_0  i.card_none_0 i.illness_t  i.health_0  i.live_alone_0 i.incomerec2_t i.psychpast_visited_0  i.antidepressants_0, family(gamma) link(log)

* Subgroup analysis

mi estimate, eform post: glm costsocietaltot_imp i.group phqdep_total_impute_t i.clinic_0 age_num_0 i.gender_0 i.education_rec_0 i.employment_0  i.card_none_0 i.illness_t  i.health_0  i.live_alone_0 i.incomerec2_t i.psychpast_visited_0  i.antidepressants_0 if risk ==1, family(gamma) link(log)
mi estimate, eform post: glm costsocietaltot_imp i.group phqdep_total_impute_t i.clinic_0 age_num_0 i.gender_0 i.education_rec_0 i.employment_0  i.card_none_0 i.illness_t  i.health_0  i.live_alone_0 i.incomerec2_t i.psychpast_visited_0  i.antidepressants_0 if risk ==2, family(gamma) link(log)
mi estimate, eform post: glm costsocietaltot_imp i.group phqdep_total_impute_t i.clinic_0 age_num_0 i.gender_0 i.education_rec_0 i.employment_0  i.card_none_0 i.illness_t  i.health_0  i.live_alone_0 i.incomerec2_t i.psychpast_visited_0  i.antidepressants_0 if risk ==3, family(gamma) link(log)

* Analysis of QALYS at 3-month follow-up (T2)

mi estimate: glm qaly_2_imp i.group AQoL8DUtility_1_imp phqdep_total_impute_t i.clinic_0 i.risk age_num_0 i.gender_0 i.education_rec_0 i.employment_0  i.card_none_0 i.illness_t  i.health_0  i.live_alone_0 i.incomerec2_t i.psychpast_visited_0  i.antidepressants_0, family(gaussian) link(identity)

* Subgroup analysis

mi estimate: glm qaly_2_imp i.group AQoL8DUtility_1_imp phqdep_total_impute_t i.clinic_0 age_num_0 i.gender_0 i.education_rec_0 i.employment_0  i.card_none_0 i.illness_t  i.health_0  i.live_alone_0 i.incomerec2_t i.psychpast_visited_0  i.antidepressants_0 if risk ==1, family(gaussian) link(identity)
mi estimate: glm qaly_2_imp i.group AQoL8DUtility_1_imp phqdep_total_impute_t i.clinic_0 age_num_0 i.gender_0 i.education_rec_0 i.employment_0  i.card_none_0 i.illness_t  i.health_0  i.live_alone_0 i.incomerec2_t i.psychpast_visited_0  i.antidepressants_0 if risk ==2, family(gaussian) link(identity)
mi estimate: glm qaly_2_imp i.group AQoL8DUtility_1_imp phqdep_total_impute_t i.clinic_0 age_num_0 i.gender_0 i.education_rec_0 i.employment_0  i.card_none_0 i.illness_t  i.health_0  i.live_alone_0 i.incomerec2_t i.psychpast_visited_0  i.antidepressants_0 if risk ==3, family(gaussian) link(identity)

* Analysis of QALYS at 12-month follow-up (T2+T3)

mi estimate: glm qalytot_imp i.group AQoL8DUtility_1_imp phqdep_total_impute_t i.clinic_0 i.risk age_num_0 i.gender_0 i.education_rec_0 i.employment_0  i.card_none_0 i.illness_t  i.health_0  i.live_alone_0 i.incomerec2_t i.psychpast_visited_0  i.antidepressants_0, family(gaussian) link(identity)

* Subgroup analysis

mi estimate: glm qalytot_imp i.group AQoL8DUtility_1_imp phqdep_total_impute_t i.clinic_0 age_num_0 i.gender_0 i.education_rec_0 i.employment_0  i.card_none_0 i.illness_t  i.health_0  i.live_alone_0 i.incomerec2_t i.psychpast_visited_0  i.antidepressants_0 if risk ==1, family(gaussian) link(identity)
mi estimate: glm qalytot_imp i.group AQoL8DUtility_1_imp phqdep_total_impute_t i.clinic_0 age_num_0 i.gender_0 i.education_rec_0 i.employment_0  i.card_none_0 i.illness_t  i.health_0  i.live_alone_0 i.incomerec2_t i.psychpast_visited_0  i.antidepressants_0 if risk ==2, family(gaussian) link(identity)
mi estimate: glm qalytot_imp i.group AQoL8DUtility_1_imp phqdep_total_impute_t i.clinic_0 age_num_0 i.gender_0 i.education_rec_0 i.employment_0  i.card_none_0 i.illness_t  i.health_0  i.live_alone_0 i.incomerec2_t i.psychpast_visited_0  i.antidepressants_0 if risk ==3, family(gaussian) link(identity)


** Clear data set from memory
```

```
clear all



******************
***************    END OF MODULE 2 - IMPUTATION ANALYSIS
************








******************
***************    START OF MODULE 3 - BOOTSTRAP ANALYSIS
************


*****************************************************************
***   TARGET_D - BOOTSTRAP WITH NESTED MULTIPLE IMPUTATION   ***
*****************************************************************

**
** The resampling method adopted in this code comprises the 'single imputation
** nested in bootstrapping' approach advocated by Brand et al., (2016) and other
** leading health economists, such as Prof Andrew Briggs. The code presented
** below involves using the MICE command to impute a singular complete dataset,
** then performing GLM analyses to examine differences in mean costs and QALYs.
**
** The margins command (dydx) is then used to estimate the marginal change in
** costs/QALYs due to a one-unit change in the intervention group (where
** 0 = control and 1 = intervention). ICERs are subsequently estimated as the
** difference in mean costs divided by the difference in mean QALYs.
**
** Brand et al., 2016 -- https://doi.org/10.1002/sim.7956
**
** NOTE: The nested bootstrap analysis has the advantage of enabling
**       the utilisation of the margins command. There is currently no
**       straightforward method by which to use the margins command alongside
**       the 'MI estimate' commands. See link below for further reading on
**       this issue.
**
** Link:
https://stats.idre.ucla.edu/stata/faq/how-can-i-get-margins-and-marginsplot-with-multiply-i
mputed-data/
**
**
** The bootstrap percentile method was used to estimate 95% confidence intervals
** (95% CIs) around the difference in mean costs, difference in mean QALYs and
** mean ICERs. This involves identifying the 2.5th and 97.5th percentiles of the
** 'm' bootstrap resamples that are generated. A total of 1,000 bootstrap resamples
** (m = 1,000) were produced.
**
**



*** PREPARATORY CODE

** Set working directory

cd "C:\Stata"


** Load dataset and keep in-scope variables

clear all
use "C:\Stata\TargetD_MERGED_inscope.dta", replace

global var_sid study_id

global var_reg group phqdep_total_impute_t clinic_0 risk /// *Regular_variables
               age_num_0 gender_0 costintv SA1_costintv SA2_costintv

global var_imp costtothealthprof_2 costtotervisit_2 /// *Imputed_variables
               costtotovnstay_2 costtotdiagtest_2 costtotmed_2 ///
               costtothealthprof_3 costtotervisit_3 costtotovnstay_3 ///
               costtotdiagtest_3 costtotmed_3 ///
               costtotprodloss_2 costtotprodloss_3 ///
```

```
               AQoL8DUtility_1 AQoL8DUtility_2 AQoL8DUtility_3 ///
               phqdep_total_impute_2 phqdep_total_impute_3

global var_adj education_rec_0 employment_0  /// *SA3_adjustment_variables
               card_none_0 illness_t  health_0  live_alone_0 ///
               incomerec2_t psychpast_visited_0  antidepressants_0

keep $var_sid $var_reg $var_imp $var_adj
order $var_sid $var_reg $var_imp $var_adj


** Duplicate existing variables for the multiple imputation analysis

qui: gen costtothealthprof_2_imp = costtothealthprof_2
qui: gen costtotervisit_2_imp = costtotervisit_2
qui: gen costtotovnstay_2_imp = costtotovnstay_2
qui: gen costtotdiagtest_2_imp = costtotdiagtest_2
qui: gen costtotmed_2_imp = costtotmed_2

qui: gen costtothealthprof_3_imp = costtothealthprof_3
qui: gen costtotervisit_3_imp = costtotervisit_3
qui: gen costtotovnstay_3_imp = costtotovnstay_3
qui: gen costtotdiagtest_3_imp = costtotdiagtest_3
qui: gen costtotmed_3_imp = costtotmed_3

qui: gen costtotprodloss_2_imp = costtotprodloss_2
qui: gen costtotprodloss_3_imp = costtotprodloss_3

qui: gen AQoL8DUtility_1_imp = AQoL8DUtility_1
qui: gen AQoL8DUtility_2_imp = AQoL8DUtility_2
qui: gen AQoL8DUtility_3_imp = AQoL8DUtility_3

qui: gen phqdep_total_impute_2_imp = phqdep_total_impute_2
qui: gen phqdep_total_impute_3_imp = phqdep_total_impute_3


** MI estimation commands

* Declare MI data set
*
* -------- IMPORTANT: DATA MUST BE SET IN THE WIDE FORMAT!! --------
* --------  BOOTSTRAP IS INCOMPATIBLE WITH THE LONG FORMAT  --------

mi set wide

* Register variables to be imputed

mi register imputed costtothealthprof_2_imp costtotervisit_2_imp costtotovnstay_2_imp ///
                    costtotdiagtest_2_imp costtotmed_2_imp costtothealthprof_3_imp ///
                    costtotervisit_3_imp costtotovnstay_3_imp costtotdiagtest_3_imp ///
                    costtotmed_3_imp costtotprodloss_2_imp costtotprodloss_3_imp ///
                    AQoL8DUtility_1_imp AQoL8DUtility_2_imp AQoL8DUtility_3_imp ///
                    phqdep_total_impute_2_imp phqdep_total_impute_3_imp

mi register regular $var_reg $var_adj

* Generate (and register) passive variables

mi passive: gen costhealthsector_2_imp = costtothealthprof_2_imp + costtotervisit_2_imp +
///
                                         costtotovnstay_2_imp + costtotdiagtest_2_imp + ///
                                         costtotmed_2_imp + costintv
mi passive: gen costhealthsector_3_imp = costtothealthprof_3_imp + costtotervisit_3_imp +
///
                                         costtotovnstay_3_imp + costtotdiagtest_3_imp + ///
                                         costtotmed_3_imp
mi passive: gen costhealthsectortot_imp = costhealthsector_2_imp + costhealthsector_3_imp

mi passive: gen costsocietal_2_imp = costhealthsector_2_imp + costtotprodloss_2_imp
mi passive: gen costsocietal_3_imp = costhealthsector_3_imp + costtotprodloss_3_imp
mi passive: gen costsocietaltot_imp = costsocietal_2_imp + costsocietal_3_imp

mi passive: gen qaly_2_imp = 0.5*(AQoL8DUtility_1_imp + AQoL8DUtility_2_imp)*(3/12)
mi passive: gen qaly_3_imp = 0.5*(AQoL8DUtility_2_imp + AQoL8DUtility_3_imp)*(9/12)
mi passive: gen qalytot_imp = qaly_2_imp + qaly_3_imp


** Save the declared multiple imputation dataset template

```

```
save "C:\Stata\TargetD_MERGED_inscope_MI_template.dta", replace



*** ESTIMATE ICERS AT 3-MONTH FOLLOW-UP (T2) AND 12-MONTH FOLLOW-UP (T2+T3)

** BS10: Adjusted - Cost per QALY | Health sector & Societal | 3 mths & 12 mths

* Load multiple imputation dataset template

clear all
use "C:\Stata\TargetD_MERGED_inscope_MI_template.dta", clear


* Declare additional covariates for GLM analyses nested within bootstrap

global var_cov phqdep_total_impute_t i.clinic_0 i.risk


* Define program

program [illegible],

    ** Preserve data environment to be restored after program termination

    preserve

    ** Perform multiple imputation

    mi impute chained ///
    (pmm, knn(10)) AQoL8DUtility_1_imp AQoL8DUtility_2_imp AQoL8DUtility_3_imp ///
                   phqdep_total_impute_2_imp phqdep_total_impute_3_imp ///
                   costtothealthprof_2_imp costtotervisit_2_imp costtotovnstay_2_imp ///
                   costtotdiagtest_2_imp costtotmed_2_imp costtothealthprof_3_imp ///
                   costtotervisit_3_imp costtotovnstay_3_imp costtotdiagtest_3_imp ///
                   costtotmed_3_imp costtotprodloss_2_imp costtotprodloss_3_imp ///
    = i.group i.gender_0 i.clinic_0 age_num_0 education_rec_0 psychpast_visited_0, ///
    by(risk) add(1) dots

    * NOTE: Do not set the seed of the nested imputation within the bootstrap.
    *       Also set the number of imputations to M=1.

    ** Replace passive variables

    mi passive: replace costhealthsector_2_imp = costtothealthprof_2_imp +
costtotervisit_2_imp + ///
                                         costtotovnstay_2_imp + costtotdiagtest_2_imp + ///
                                         costtotmed_2_imp + costintv
    mi passive: replace costhealthsector_3_imp = costtothealthprof_3_imp +
costtotervisit_3_imp + ///
                                         costtotovnstay_3_imp + costtotdiagtest_3_imp + ///
                                         costtotmed_3_imp
    mi passive: replace costhealthsectortot_imp = costhealthsector_2_imp +
costhealthsector_3_imp

    mi passive: replace costsocietal_2_imp = costhealthsector_2_imp + costtotprodloss_2_imp
    mi passive: replace costsocietal_3_imp = costhealthsector_3_imp + costtotprodloss_3_imp
    mi passive: replace costsocietaltot_imp = costsocietal_2_imp + costsocietal_3_imp

    mi passive: replace qaly_2_imp = 0.5*(AQoL8DUtility_1_imp + AQoL8DUtility_2_imp)*(3/12)
    mi passive: replace qaly_3_imp = 0.5*(AQoL8DUtility_2_imp + AQoL8DUtility_3_imp)*(9/12)
    mi passive: replace qalytot_imp = qaly_2_imp + qaly_3_imp

    ** Extract single imputed dataset

    mi extract 1, clear


    ** Conduct GLM analysis using single MI dataset in memory

    * GLM analysis of QALY variables - 3 months

    glm qaly_2_imp i.group AQoL8DUtility_1_imp $var_cov, family(gaussian) link(identity)

    margins, dydx(group)
    return scalar q1 = el(r(table),1,2)

    * GLM analysis of QALY variables - 12 months

```

```
    glm qalytot_imp i.group AQoL8DUtility_1_imp $var_cov, family(gaussian) link(identity)

    margins, dydx(group)
    return scalar q6 = el(r(table),1,2)

    * GLM analysis of Cost variables - 3 months, Health sector

    glm costhealthsector_2_imp i.group $var_cov, family(gamma) link(log) eform

    margins, dydx(group)
    return scalar c1 = el(r(table),1,2)

    * GLM analysis of Cost variables - 3 months, Societal

    glm costsocietal_2_imp i.group $var_cov, family(gamma) link(log) eform

    margins, dydx(group)
    return scalar c2 = el(r(table),1,2)

    * GLM analysis of Cost variables - 12 months, Health sector

    glm costhealthsectortot_imp i.group $var_cov, family(gamma) link(log) eform

    margins, dydx(group)
    return scalar c6 = el(r(table),1,2)

    * GLM analysis of Cost variables - 12 months, Societal

    glm costsocietaltot_imp i.group $var_cov, family(gamma) link(log) eform

    margins, dydx(group)
    return scalar c7 = el(r(table),1,2)

end

* Perform bootstrap analysis

set seed 19

bootstrap inc_qalys_3m=r(q1)  inc_costs_hlth_3m=r(c1)  inc_costs_sctl_3m=r(c2)  ///
          inc_qalys_12m=r(q6) inc_costs_hlth_12m=r(c6) inc_costs_sctl_12m=r(c7), ///
          reps(10) strata(group) saving(TargetD_Bootstrap_GLM_BS10, replace) : myBS10

use TargetD_Bootstrap_GLM_BS10, clear
estat bootstrap, all



******************
***************    END OF MODULE 3 - BOOTSTRAP ANALYSIS
************
```
