# Supplementary material for: Economic evaluation of the Target-D platform to match depression management to severity prognosis in primary care: A within-trial cost-utility analysis
Source: PLoS One. 2022 May 25;17(5):e0268948. doi: 10.1371/journal.pone.0268948 (PMC9132336; doi:10.1371/journal.pone.0268948)
Supplement: Attachment — Submitted filename: 00b_Target_D_Response_to_Reviewers.pdf [file pone.0268948.s003.pdf]

Editor #1:

E1.1 – 1. Please ensure that your manuscript meets PLOS ONE's style requirements, including those for file naming. The PLOS ONE style templates can be found at
https://journals.plos.org/plosone/s/file?id=wjVg/PLOSOne_formatting_sample_main_body.pdf
and
https://journals.plos.org/plosone/s/file?id=ba62/PLOSOne_formatting_sample_title_authors_affiliations.pdf

We have sought to comply with the PLOS ONE style templates the best we can.

E1.2 – 2. Please update your submission to use the PLOS LaTeX template. The template and more information on our requirements for LaTeX submissions can be found at
http://journals.plos.org/plosone/s/latex.

For now, we have opted to submit our manuscript using Word document files. We will be more than willing to provide a submission in the PLOS LaTeX template following the acceptance of our paper.

E1.3 – 3. Thank you for stating the following in the Acknowledgments Section of your manuscript: "The authors would like to thank all the patients, family physicians, and clinics who took part in Target-D ... Finally, we thank staff at the former Melbourne Networked Society Institute (MNSI) who were funded to build the Target-D website." We note that you have provided funding information that is not currently declared in your Funding Statement. However, funding information should not appear in the Acknowledgments section or other areas of your manuscript. We will only publish funding information present in the Funding Statement section of the online submission form. Please remove any funding-related text from the manuscript and let us know how you would like to update your Funding Statement. Please include your amended statements within your cover letter; we will change the online submission form on your behalf.

We have modified the acknowledgements to remove all references to funding information. Please see the amended text below.

> *The authors would like to thank all the patients, family physicians, and clinics who took part in Target-D; and the many research assistants who assisted with data collection. The data used to develop the clinical prediction tool were collected as a part of the diamond project (NHMRC project ID: 299869, 454463, 566511 and 1002908). We acknowledge the 30 dedicated family physicians, their patients, and clinic staff for making the diamond study possible. We also acknowledge staff and students at the School of Computing and Information Systems at the University of Melbourne for early work that informed the presentation of the e-health platform as well as the focus group participants that provided feedback on early versions of the Target-D materials. Finally,*

we thank staff at the former Melbourne Networked Society Institute (MNSI) who built the Target-D website.

E1.4 – 4. In your Data Availability statement, you have not specified where the minimal data set underlying the results described in your manuscript can be found. PLOS defines a study's minimal data set as the underlying data used to reach the conclusions drawn in the manuscript and any additional data required to replicate the reported study findings in their entirety. All PLOS journals require that the minimal data set be made fully available. For more information about our data policy, please see http://journals.plos.org/plosone/s/data-availability. Upon re-submitting your revised manuscript, please upload your study's minimal underlying data set as either Supporting Information files or to a stable, public repository and include the relevant URLs, DOIs, or accession numbers within your revised cover letter. For a list of acceptable repositories, please see http://journals.plos.org/plosone/s/data-availability#loc-recommended-repositories. Any potentially identifying patient information must be fully anonymized. Important: If there are ethical or legal restrictions to sharing your data publicly, please explain these restrictions in detail. Please see our guidelines for more information on what we consider unacceptable restrictions to publicly sharing data: http://journals.plos.org/plosone/s/data-availability#loc-unacceptable-data-access-restrictions. Note that it is not acceptable for the authors to be the sole named individuals responsible for ensuring data access. We will update your Data Availability statement to reflect the information you provide in your cover letter.

5. We note that you have indicated that data from this study are available upon request. PLOS only allows data to be available upon request if there are legal or ethical restrictions on sharing data publicly. For more information on unacceptable data access restrictions, please see http://journals.plos.org/plosone/s/data-availability#loc-unacceptable-data-access-restrictions.
In your revised cover letter, please address the following prompts:
a) If there are ethical or legal restrictions on sharing a de-identified data set, please explain them in detail (e.g., data contain potentially sensitive information, data are owned by a third-party organization, etc.) and who has imposed them (e.g., an ethics committee). Please also provide contact information for a data access committee, ethics committee, or other institutional body to which data requests may be sent.
b) If there are no restrictions, please upload the minimal anonymized data set necessary to replicate your study findings as either Supporting Information files or to a stable, public repository and provide us with the relevant URLs, DOIs, or accession numbers. For a list of acceptable repositories, please see http://journals.plos.org/plosone/s/data-availability#loc-recommended-repositories.
We will update your Data Availability statement on your behalf to reflect the information you provide.

Ethics approval for the clinical trial underlying the submitted manuscript was obtained through the University of Melbourne. We have contacted Ms Hilary Young, Secretary for the Medicine and Dentistry Human Ethics Sub-Committee (HESC) at the University of Melbourne, to provide us with guidance on this matter (*Phone: +61 3 8344 8595, Email: hilary.young@unimelb.edu.au*). We have attached a copy of the resulting correspondence. To summarise, we have been notified that the plain language statement

of the original study does not provide a contingency for study participants to provide informed consent for any prospective data sharing, particularly given that collected data is potentially identifying and involves sensitive personal information. As such, we are unable to release the data as part of any minimum dataset. We have amended our Data Availability Statement to read:

> *Ethical restrictions prevent the sharing of potentially sensitive data provided by study participants over the course of the Target-D clinical trial (Australian New Zealand Clinical Trials Registry ACTRN12616000537459). No contingency was included in the original plain language statement for study participants to provide informed consent to any prospective sharing of their personal data, whether as part of a minimum dataset or another form. For all enquiries regarding the Target-D clinical trial and the underlying dataset, please contact the chief investigator, Prof Jane Gunn (j.gunn@unimelb.edu.au). For general enquiries regarding the ethics approval of the trial, please contact the Office of Research Ethics and Integrity (OREI) at The University of Melbourne (HumanEthics-Enquiries@unimelb.edu.au).*

As a compromise to our inability to provide a minimum dataset, we have now included an additional supplementary appendix (S2 Appendix) that contains summary metadata on all in-scope data variables, alongside a copy of the Stata do-file. The following sentence has been added to the end of the 'Statistical analysis' section of the Methods in lines 175-176:

> *A summary list of all variables included in the statistical analysis is provided in S2 Appendix, alongside the Stata do-file used to implement the statistical analysis.*

Reviewer #1:

The paper aims to assess cost-effectiveness of the target-D intervention against usual care. Mental health and primary care are both very important subjects for public health. Evaluation of e-health is also of prime importance. The work is original but more methodological precisions were needed.
Main concerns:

R1.1 – 1) A micro-costing approach was used to estimate the cost of the target-D intervention except for the clinician-guided iCBT program (moderate prognostic group) (page 5, lines 112-113: "The average cost between two Australia clinician-guides iCBT programs was applied to participants in the moderate prognostic group"). Why?

There is no definitive, gold standard unit cost for clinician-guided iCBT in Australia. Instead, we have available two alternative unit costs that provide a low-to-high range of possible values. The point value used in the base case analysis comprised the average of the low and high values. This is the rationale for why we performed a subsequent sensitivity analysis analysing the impact of adopting the highest unit cost ($222 per person). In response to this comment, we have added the following sentence to improve clarity on lines 116-117:

> *In this instance, the two programs represent a low-to-high range of possible unit cost values for clinician-guided iCBT in Australia. A subsequent sensitivity analysis was done to test the impact of using the highest unit cost, rather than the average.*

R1.2 – 2) Some costs appeared to be research-induced (e.g. p5, lines 105-106 "personnel time to approach individuals in the GP waiting room involved one minute per encounter"). Please clarify

We made sure to include costs that would occur in routine practice and to exclude research-related costs. Resource use involving the research assistants (e.g., approaching individuals in the GP waiting room or periodic check-in phone calls) will need to be performed by similarly qualified staff if the intervention were to be implemented as part of routine practice. We have added a sentence to lines 120-122 to clarify this point:

> *It is anticipated that all costed activities described above that involve research assistants will likely require similarly qualified staff to facilitate the implementation of the intervention as part of routine practice.*

R1.3 – 3) The drop-out rate was very important for the RUQ data and the AQoL-8D data. Not enough information is given to the lector on how the missing data were treated. Bootstrapped data need to be stratified by treatment arm. The process of missing data need to be tested, not assumed (page 6, line 140: "assuming data were missing at random"). Tests should be presented and discussed. If data were not MAR, extensive sensitivity analyses (scenarios) on costs and quality-of-life utilities need to be

conducted (not only complete case analysis, valid if and only if missing data were MCAR). Please give reference if this issue was treated in a previous paper.

We thank the reviewer for encouraging us to be clear in how we have chosen to address the problem of missing data. In response to this comment, we have now added a new section to the supplementary materials, 'Supplementary Text S5. Analysis of missing data mechanisms'. In this supplement, we have provided a detailed exploration of missing data patterns and an empirical rationale for why we have concluded that there is sufficient evidence to infer that missing utility/cost data can be considered missing at random (as opposed to missing not at random). Based on these analyses, we identified several baseline sociodemographic variables that were associated with the likelihood of missing utility/cost values. We have consequently included these variables as adjustment covariates in the multiple imputation analysis, which was also updated. All results based on these methodological refinements have been amended accordingly.

We have modified text on lines 144-150 to read:

> *Multiple imputation methods were implemented in Stata to account for missing data that were deemed missing at random following several exploratory analyses presented in Supplementary Text 5 in S1 Appendix. Missing cost and outcomes data were imputed 100 times using multiple imputation by chained equations (MICE), with predictive mean matching and adjustment for baseline covariates associated with data missingness – i.e., trial arm, clinic, age, gender, highest level of education and having visited a psychologist/counsellor in the past 12 months.*

R1.4 – 4) Two GLM had been estimated, one for costs (link=log; family=gamma) and another for utility scores (link=identity; family=Gaussian). In the paper, it is not clear if these models were re-estimated for each bootstrapped sample or only once. If ICER is computed from estimated coefficients, how the ratio was converted into a difference for costs? How potential correlation between errors terms of the QALY equation and the cost equation were taken into account in the analysis?

The reviewer is justified in their call for further descriptive detail on the methods used to implement bootstrapping. We confirm that the ICER was computed using estimated GLM coefficients of the difference in mean costs and the difference in mean QALYs. In the original analysis, we adopted a resampling method that encompassed, 'bootstrapping nested in multiple imputation'. Since then, we have encountered recommendations by Brand et al., 2019 (doi: 10.1002/sim.7956) and Prof Andy Briggs who collectively advocate, 'single imputation nested in bootstrapping'. Based on these recommendations, we have revised our analytic approach and modified our description of the methods/results accordingly.

The text on lines 161-173 has now been amended to read:

> *Incremental cost-effectiveness ratios (ICERs) were calculated as the difference in mean costs between the intervention and control arms divided by the difference in mean QALYs. ICERs were calculated by study perspective (health sector and societal), follow-up period (3 and 12 months) and, for the subgroup analysis, by prognostic group (total, minimal/mild, moderate and severe). A resampling method comprising single imputation nested in bootstrapping [17] was used to quantify the impact of input parameter uncertainty around the resulting differences in mean costs/QALYs and the mean ICERs. This method works by generating a single call to the MICE procedure to produce a complete dataset with which to analyse GLMs of costs/QALYs within each bootstrap resample. Following the generation of 1,000 bootstrap resamples, the bootstrap percentile method was used to estimate 95% confidence intervals (95% CI) around the differences in mean costs/QALYs and the mean ICERs [18]. The intervention was considered cost-effective if the resulting ICER was less than the Australian willingness-to-pay threshold of A$50,000 per QALY [19-21].*

Furthermore, we have now included an additional supplementary appendix (S2 Appendix) that contains both the Stata do-file and a summary list of data variables. The following sentence has been added to the end of the 'Statistical analysis' section of the Methods in lines 175-176:

> *A summary list of all variables included in the statistical analysis is provided in S2 Appendix, alongside the Stata do-file used to implement the statistical analysis.*

R1.5 – 5) Concerning the QALY equation, was the value at baseline systematically included among covariates? (not clear page 6, lines 147-149: "All GLM models were estimated with and without adjustment for several baselines specified in the study protocol- i.e. baseline PHQ-9 score (not QALY, as requested in guidelines),general practice and prognostic group")

The reviewer has made an important critique of our methods. Our initial analysis did not adjust for baseline utility scores derived using the AQoL-8D measure as we were narrowly focussed on reproducing the primary outcomes analysis, which made adjustments for the baseline PHQ-9 score. Moreover, we had *a priori* postulated that baseline PHQ-9 scores would be (in theory) highly correlated to baseline AQoL-8D scores. Following the reviewer's comment, we have made a decision to re-analyse QALY outcomes after making an additional adjustment for baseline AQoL-8D scores.

In the methods, we have amended lines 155-159:

> *All GLMs were estimated with and without adjustment for several baseline covariates specified in the study protocol – i.e., baseline PHQ-9 score, general practice and prognostic group [7]. Baseline AQoL-8D scores were also included as an additional baseline covariate for GLMs involving QALY outcomes.*

The results that are reported in Table 3 now reflect these changes. Additionally, the first footnote to the results presented in Table 3 on line 253 has also been amended to reflect the addition of the baseline AQoL-8D score as a baseline covariate.

R1.6 – 6) It could be interesting to present details on cost provided by microcosting for each level of intervention. Page 8, lines 191-194, be more affirmative "This was likely due to the high-cost nature of collaborative care delivered to participants in the severe group".

Detailed costs encompassing the microcosting approach are presented for each intervention level in Supplementary Table S3. We have amended to sentence on lines 212-214 to read:

> *This was likely due to the high-cost nature of collaborative care delivered to participants in the severe group (see Supplementary Table 3 in S1 Appendix for detailed costs).*

R1.7 – 7) Acceptability curves could be estimated for the 3 prognostic groups.

We appreciate the reviewer's suggestion here. However, we have opted not to present in-depth results for the three prognostic groups (i.e., cost-effectiveness planes or cost-effectiveness acceptability curves) given that these are subgroup analyses that are underpowered to detect statistically significant differences, particularly when compared to the aggregate findings. We have amended text in lines 162-165 to emphasise the fact that the analysis of prognostic groups encompasses a subgroup analysis:

> *ICERs were calculated by study perspective (health sector and societal), follow-up period (3 and 12 months) and, for the subgroup analysis, by prognostic group (total, minimal/mild, moderate and severe).*

R1.8 – 8) The conclusion (page 19, line 338-341) was very strong, not really in line with methodological issues mentioned page 18, lines 318-319 and 325-327

We have amended the relevant texts on lines 359-362 and lines 365-366 to soften conclusions drawn based on our study findings. These texts now read as follows:

> *The results of this study suggest that stepped care may be dominant when compared to usual care. Additionally, study findings appear to support the existing literature by suggesting that stepped care for depression can deliver improved clinical outcomes without increasing costs.*
>
> *The Target-D intervention is likely to represent good value for money and provides indicative support for further development of digitally supported mental health care.*

Reviewer #2:

The authors present an economic evaluation of the Target-D intervention, based on resource utilization information collected during a clinical trial of Target-D versus usual care in Melbourne, Australia. Results are presented both from a health sector perspective and a societal perspective. Authors conclude that Target-D likely has good value for health care decision makers. The manuscript is well written. I only have a few minor recommendations for the authors.

R2.1 – 1. line 43: authors state that health sector and societal costs were "comparable" between trial arms at 3 and 12 months. Authors should replace "comparable" with "not significantly different" since authors did not do a specific test for equality of the costs.

We thank the reviewer for this suggestion and have amended the Abstract text accordingly (see line 43).

R2.2 – 2. Authors should provide the number of control and intervention participants in each of the prognostic groups (minimal/mild, moderate, severe), rather than relying on readers to go to the published paper on trial results to get this information. This can likely just be put in the text in lines 177-178.

We have added this information to lines 194-198 at the beginning of the Results section, as requested by the reviewer.

R2.3 – 3. lines 208 (note under Table 1), 217 (note under Table 2), and 235 (note under Table 3): "partcipants" should be "participants"

We thank the reviewer for spotting this mistake. The spelling of this word has now been corrected in all relevant locations.
