# Supplementary material for: Economic evaluation of the Target-D platform to match depression management to severity prognosis in primary care: A within-trial cost-utility analysis
Source: PLoS One. 2022 May 25;17(5):e0268948. doi: 10.1371/journal.pone.0268948 (PMC9132336; doi:10.1371/journal.pone.0268948)
Supplement: Attachment — Submitted filename: 00b_Target_D_Response_to_Reviewers.pdf [file pone.0268948.s004.pdf]

## Reviewer #1:

Thank you to the authors for adressing all my comments on the previous version of the paper. I have two (marginal) left comments :

**R1.1** – 1) As the effectiveness difference between arms is very small (non significant), I should be preferable to estimate the 95% CI for ICER using the Fieller's method instead of the bootstrap method. The former is less sensitive to misinterpretation of the CI bounds than the latter [ see https://www.iresp.net/wp-content/uploads/2018/12/Siani-article-3.pdf ]

We thank the reviewer for suggesting Fieller's theorem as a potentially more accurate method by which to estimate the 95% confidence interval for the mean ICER; under the scenario when the expected value of the difference in effectiveness outcomes between trial arms approaches zero (as occurs in our study). We have read through the article by Siani et al. (2003) and note that: (1) Fieller's theorem has the potential to produce more accurate 95% confidence interval bounds with excellent coverage over the 95% confidence region - based largely on simulations analysed in the quoted study; and (2) 95% CIs produced using the non-parametric bootstrap percentile method can potentially lead to confidence bounds with a marginally higher coverage of the target 95% confidence region (i.e., 97% coverage of the mean ICER). We conducted a quick search of the literature and struggled to find studies that have replicated the findings of Siani et al., (2003). This makes it difficult to confirm the veracity of the phenomena identified by Siani et al. (2003). Even so, we concede that the potential for the bias identified by Siani et al. (2003) remains.

If the aforementioned bias were to transpire, then we contend that such imprecision in the estimation of 95% confidence bounds will not have a material impact on the interpretation of our study findings. This is due largely to the bootstrap resampling results which indicated a high degree of uncertainty around the expected value of mean ICERs estimated across all base case and subgroup analyses (i.e., bootstrap resamples for mean ICERs consistently covered all four quadrants of the cost-effectiveness plane). As such, the 95% confidence bounds produced by the bootstrap percentile method did not approach the nominated WTP threshold of A$50,000 per QALY. This was especially true when the lower and upper 95% confidence bounds spanned the South-East ('dominant') and North-West ('dominated') quadrants of the cost-effectiveness plane. In summary, any (comparatively small) imprecision around the 95% confidence bounds presented in Table 4 is expected to be inconsequential when compared to the extreme range of lower and upper 95% confidence bounds.

In response to this comment, we have added a note to Table 4 stating that:

> *The mean difference in QALYs between trial arms was observed to approach zero across all base case and subgroup analyses. This can potentially lead to the lower and upper bounds of a 95% confidence interval, derived using the bootstrap percentile method, encompassing a marginally higher coverage than the target 95% confidence region (e.g.,*

*97% coverage of the mean ICER) [22]. Even so, any resulting imprecision in the estimation of the 95% confidence bounds will likely be inconsequential to the interpretation of study findings given the wide range of ICER values that were consistently observed between the lower and upper confidence bounds (e.g., confidence bounds ranging between 'dominant' and 'dominated'). This reflects the high degree of uncertainty observed across mean ICER values for all base case and subgroup analyses; with bootstrap resamples consistently spanning all four quadrants of the cost-effectiveness plane.*

**R1.2 –** 2) In the QALY equation, the utility score at inclusion should be included as covariate (not baseline AQoL-8D score) [see Willan and Briggs, Statistical analysis of cost-effectiveness data, Statistics in Practice, Wiley, page 24-25]

We apologise to the reviewer for using imprecise terminology that has, in turn, led to this instance of semantic confusion. When we used the term 'baseline AQoL-8D score', our intended meaning was 'baseline AQoL-8D utility weight' – i.e., utility weights estimated based on scoring the AQoL-8D multi-attribute utility instrument. In response to this comment, we have changed all instances of 'AQoL-8D score(s)' to 'AQoL-8D utility weight(s)'.
